# Understanding Cognitive Aging Through White Matter: A Fixel‐Based Analysis

**DOI:** 10.1002/hbm.70121

**Published:** 2024-12-25

**Authors:** Emma M. Tinney, Aaron E. L. Warren, Meishan Ai, Timothy P. Morris, Amanda O'Brien, Hannah Odom, Bradley P. Sutton, Shivangi Jain, Chaeryon Kang, Haiqing Huang, Lu Wan, Lauren Oberlin, Jeffrey M. Burns, Eric D. Vidoni, Edward McAuley, Arthur F. Kramer, Kirk I. Erickson, Charles H. Hillman

**Affiliations:** ^1^ Department of Psychology Northeastern University Boston Massachusetts USA; ^2^ Center for Cognitive & Brain Health Northeastern University Boston Massachusetts USA; ^3^ Department of Neurosurgery, Brigham and Women's Hospital Harvard Medical School Boston Massachusetts USA; ^4^ Department of Physical Therapy, Movement, & Rehabilitation Sciences Northeastern University Boston Massachusetts USA; ^5^ Department of Applied Psychology Northeastern University Boston Massachusetts USA; ^6^ Beckman Institute University of Illinois Urbana Illinois USA; ^7^ Department of Bioengineering University of Illinois Urbana Illinois USA; ^8^ AdventHealth Research Institute Neuroscience Orlando Florida USA; ^9^ Department of Biostatistics University of Pittsburgh Pittsburgh Pennsylvania USA; ^10^ Weill Cornell Institute of Geriatric Psychiatry Weill Cornell Medicine White Plains New York USA; ^11^ University of Kansas Medical Center Kansas City Kansas USA; ^12^ Department of Health and Kinesiology University of Illinois Urbana Illinois USA

**Keywords:** aging, cognitive decline, DWI, fixel‐based analysis, white matter

## Abstract

Diffusion‐weighted imaging (DWI) has been frequently used to examine age‐related deterioration of white matter microstructure and its relationship to cognitive decline. However, typical tensor‐based analytical approaches are often difficult to interpret due to the challenge of decomposing and (mis)interpreting the impact of crossing fibers within a voxel. We hypothesized that a novel analytical approach capable of resolving fiber‐specific changes within each voxel (i.e., fixel‐based analysis [FBA])—would show greater sensitivity relative to the traditional tensor‐based approach for assessing relationships between white matter microstructure, age, and cognitive performance. To test our hypothesis, we studied 636 cognitively normal adults aged 65–80 years (mean age = 69.8 years; 71% female) using diffusion‐weighted MRI. We analyzed fixels (i.e., fiber‐bundle elements) to test our hypotheses. A fixel provides insight into the structural integrity of individual fiber populations in each voxel in the presence of multiple crossing fiber pathways, allowing for potentially increased specificity over other diffusion measures. Linear regression was used to investigate associations between each of three fixel metrics (fiber density, cross‐section, and density × cross‐section) with age and cognitive performance. We then compared and contrasted the FBA results to a traditional tensor‐based approach examining voxel‐wise fractional anisotropy. In a whole‐brain analysis, significant associations were found between fixel‐based metrics and age after adjustments for sex, education, total brain volume, site, and race. We found that increasing age was associated with decreased fiber density and cross‐section, namely in the fornix, striatal, and thalamic pathways. Further analysis revealed that lower fiber density and cross‐section were associated with poorer performance in measuring processing speed and attentional control. In contrast, the tensor‐based analysis failed to detect any white matter tracts significantly associated with age or cognition. Taken together, these results suggest that FBAs of DWI data may be more sensitive for detecting age‐related white matter changes in an older adult population and can uncover potentially clinically important associations with cognitive performance.

## Graphical abstract

We assessed the sensitivity and utility of a fixel‐based white matter analysis to detect age‐related white matter degeneration. Results revealed that fixel‐based analyses were more sensitive than tensor‐based analyses and that age‐related white matter decline was related to cognitive decline.

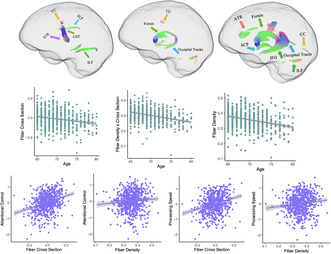

## 1 Introduction

Aging is associated with extensive white matter changes (Liu et al. 2017; Madden et al. 2012), which are, in turn, associated with age‐related cognitive decline (Charlton et al. 2006; DeCarli et al. 1995; Turken et al. 2008). Encompassing almost half of cerebral volume, white matter comprises myelinated axons, unmyelinated axons, and myelin‐producing glial cells (Zhang and Sejnowski 2000). These myelinated axons are responsible for facilitating efficient neurotransmission between subcortical and cortical areas, occupying the majority of the space in white matter (Wang et al. 2008). With advancing age, demyelination—a process whereby the protective myelin sheath around axons deteriorates—occurs, leading to disruptions in neurotransmitter efficiency and subsequent white matter degeneration and cognitive decline.

Characterization of white matter integrity can be performed noninvasively using diffusion‐weighted imaging (DWI) (Basser and Pierpaoli 1996). DWI captures the movement of water molecules and can be used to probe the microstructural integrity and composition of brain tissue. In white matter, water molecules are typically diffused in the direction parallel to the long axis of the axon bundles. Traditionally, diffusivity within a given voxel has been modeled using tensor‐based approaches (Basser and Pierpaoli 1998), which provide measures of the relative directionality (fractional anisotropy [FA]) and rate (mean diffusivity [MD]) of water diffusion as an indirect assessment of white matter integrity. In addition, measures can be calculated according to the rate of diffusion along the primary diffusion axis (axial diffusivity [AD]) and are thought to be representative of axonal injury (Song et al. 2003). Typically, aging across the lifespan and cognitive decline are associated with decreasing FA and increasing MD and AD (Bender, Völkle, and Raz 2016; Charlton et al. 2006; Coelho et al. 2021; Sexton et al. 2014; Tinney et al. 2023). However, age‐related white matter degeneration has also been found to demonstrate inconsistent results (Kumar et al. 2013; Mendez Colmenares et al. 2023; Oschwald et al. 2021). This inconsistency between studies may suggest that tensor‐derived, voxel‐based metrics may not fully capture the extent and specificity of age‐related changes to white matter pathways.

Prior work in the field of aging and DWI has limitations in the analysis approach that do not accommodate complex white matter fiber architectures. Traditional tensor‐based analyses are unable to model the presence of multiple crossing fiber pathways within a single voxel, which is found in most if not all, white matter voxels (Jeurissen et al. 2012). Tensor‐based metrics are inherently nonspecific to axonal properties, and metrics can be misleading due to signal contamination outside of the axons (Beaulieu 2014; Dhollander et al. 2021). Prior work comparing tensor‐ and fixel‐based metrics conclude that these tensor‐based metrics lack specificity due to macrostructural complexity and, therefore, are not reflective of microstructure (Grazioplene et al. 2018; Mito et al. 2018). Tensor‐based metrics represent an average across each voxel and are thus insensitive to subvoxel, fiber‐specific changes that may be associated with aging and cognition.

The present work utilizes a recently developed analysis approach based on “fixels”—fiber‐bundle elements—to investigate white matter fiber‐specific associations with age and cognition in healthy older adults. The fixel‐based analysis (FBA) framework can be used to characterize each fixel by three metrics; (i) *fiber density* (FD), a measure of white matter microstructure thought to be proportional to the total intra‐axonal volume of axons aligning with a specific fiber population; (ii) *fiber cross‐section* (FC), reflecting macroscopic fiber‐bundle morphology; and (iii) *fiber density and cross‐section* (FDC), calculated as the product of FD and FC, providing a measure sensitive to both intra‐axonal volume and cross‐sectional size of a specific fiber bundle (Dhollander et al. 2021). FBA provides insight into individual fiber populations in the presence of crossing fiber pathways, thus potentially offering sensitive measures of fiber packing, connection efficiency, and tract coherence, facilitating a deeper understanding of white matter health (D. Raffelt et al. 2012; D. A. Raffelt et al. 2017) as compared to traditional analytical approaches.

Several studies have previously reported both negative and positive relationships between fixel metrics and age (Choy et al. 2020; Han et al. 2023; Kelley et al. 2021; Zivari Adab et al. 2020). However, these prior studies focused on lifespan trajectories instead of solely older adults, which may help to narrow the age range in which the rate and extent of deterioration are clinically important to detect. In addition, prior research has not examined whether FBA outcomes are indeed more sensitive than tensor‐based analyses for assessing white matter degeneration with age. Finally, prior work has not examined the cognitive and clinical relevance of FBA associations with age—that is, whether age‐related variation in FBA metrics is associated with cognitive performance.

Here, we conducted whole‐brain, cross‐sectional FBA and tensor‐based analyses to examine the sensitivity of each approach for detecting associations with age in 636 cognitively normal older adults using baseline data from a multisite randomized controlled trial. Furthermore, we examined whether any white matter associations with age could be detected using traditional tensor‐based methods (i.e., equivalent sensitivity) or whether the fixel analysis would be superior at detecting associations with age. We examined the associations between age‐related white matter degeneration and cognitive decline by examining associations between the FBA metrics and cognitive performance. Finally, we examined moderating factors (APOE4, white matter hyperintensities [WMH], and sex) of the relationship between age and FBA metrics. We hypothesized that increasing age would be associated with reduced white matter integrity in all FBA metrics and that reduced FBA metrics would, in turn, be associated with poorer cognitive performance. Second, we hypothesized that whole‐brain FBA would show greater sensitivity to age‐related deterioration in participants older than 65 years old as compared to a traditional tensor‐based analysis approach.

## 2 Methods

### 2.1 Participants

Participants were recruited as part of the Investigating Gains in Neurocognition in an Intervention Trial of Exercise (IGNITE) study (Erickson, Grove et al. 2019). A total of 648 cognitively unimpaired participants met the inclusion criteria and were enrolled in the study. Participants were between the ages of 65–80 and were recruited in equal numbers across three different sites (University of Pittsburgh, Northeastern University, and University of Kansas, USA). Of the 648 participants, 636 had DWI data available for the current analysis. Table 1 shows the participant demographics. Briefly, inclusion criteria included individuals who were not engaging in > 20 min of structured exercise on 3 or more days per week, English‐speaking, cognitively normal, no prior diagnosis of dementia, Parkinson's disease or other neurological condition, MRI‐compatible, and safe to engage in regular moderate‐intensity exercise (see Erickson et al. 2019 for a detailed description of the study protocol).

**TABLE 1:** Participant characteristics.

	With DWI (*n* = 636)	Total (*n* = 648)
Age (mean, SD, range)	69.8 (3.73) [65–80]	69.9 (3.75) [65–80]
Sex	71% Female, 29% male	71% Female, 29% male
Race		
Black/African American	19%	19%
Asian	1.6%	1.5%
Caucasian/White	75.7%	75.8%
Native Hawaiian/Pacific Islander	0.3%	0.3%
Multiracial	2.5%	2.5%
Another racial identity	0.9%	0.9%
Years of education (mean, SD, range)	16.31 (2.21) [10–20]	16.32 (2.21) [10–20]
APOE4+	28%	28%

### 2.2 Cognitive Testing

Participants completed a battery of cognitive and neuropsychological tests, including measures of processing speed (Salthouse 2000) (Letter Comparison Test, Digit Symbol Substitution Test, Trail Making Test, Part A), working memory (Charlton et al. 2010) (N‐Back Working Memory Task, Spatial Working Memory Task, List Sorting Working Memory Task), visuospatial processing (Libon et al. 1994) (Matrix Reasoning, Spatial Relations, Clock Draw), episodic memory (Tromp et al. 2015) (Brief Visuospatial Memory Test, Picture Sequencing Test, Hopkins Verbal Learning Test, Logical Memory Task, Verbal Paired Associates), and attentional control (Coubard et al. 2011) (Flanker Task, Stroop Task (incongruent trial), Dimensional Change Card Sort task, Trail Making Test, Part B). Cognitive testing was administered by certified psychometricians and completed over 2 days. All cognitive tests have established validity and reliability in aging samples (Erickson, Grove et al. 2019). Details of the cognitive battery and confirmatory factor analysis (CFA) to reduce the dimensionality of the data have been previously described (Oberlin et al. in press). The composite scores resulting from this analysis were used as outcomes in the current analysis. The five domains (factors) identified by the CFA were: (i) working memory, referring to the temporary storage and manipulation of limited amounts of information; (ii) attentional control, the ability to focus and shift attention in a flexible way; (iii) visuospatial function, the ability to retain and process an object's identity and spatial location; (iv) processing speed, the speed with which the brain receives, understands, and responds to information; and (v) episodic memory, the capacity to recall past experiences. For each domain, higher scores indicate better performance. While we performed comprehensive neuropsychological testing with consensus adjudication for those with mild cognitive impairment (MCI) and dementia, given known limitations of neuropsychological testing and evaluation and the often variable definitions of MCI, it is possible that there were participants that were included that could be near the MCI range. More specifically, the Telephone Interview for Cognitive Status (TICS) (< 25) was used to screen for possible MCI or dementia in the prescreening phone call and a clinical adjudication of the battery of tests was used to determine MCI status. However, we intentionally recruited participants with a range of baseline cognitive performance so that the study was not hampered by superior or above‐average cognitive performance or ceiling levels of performance. Thus, we intend to indicate that some participants at the lower end of the cognitive performance spectrum met eligibility criteria, but are still considered low‐performing participants.

### 2.3 MRI Data Acquisition and Initial Preprocessing

The University of Pittsburgh and Northeastern University used a Siemens Prisma 3T scanner with a 64‐channel head coil and the University of Kansas Medical Center used a Siemens Skyra 3T scanner with a 32‐channel head coil. The MRI protocol and imaging quality were standardized across sites before data collection began to ensure uniformity of procedures and sequences across data collection sites and scanners. Scan quality was examined by phantom scanning on a monthly basis throughout the study to ensure stability of the sequences and scanners and weekly quality assurance checks were conducted. A human phantom was also scanned annually at each site to ensure standardization and image quality. The acquisition protocol included a high‐resolution structural 3D magnetization prepared rapid acquisition with gradient echo (MPRAGE) T1‐weighted anatomical scan (0.8 mm isotropic resolution, TE/TI/TR = 2.31/1060/2400 ms, field of view 256 mm, 224 slices) and a multi‐shell DWI scan (resolution: 2.5 × 2.5 × 2.5 mm, TE/TR = 95.6/2800 ms, multiband factor = 4, and *b* values of 1500 and 3000 s/mm^2^ acquired with 64 gradient directions in each shell). Intracranial volume was estimated using the T1‐weighted scans segmented by the “recon‐all” pipeline in FreeSurfer version 6.0 (Collins et al. 1994). Each DWI scan first underwent minimal preprocessing steps, including head motion, field map, and eddy current corrections prior to analysis (Andersson, Skare, and Ashburner 2003; Jenkinson et al. 2012; S. M. Smith et al. 2004). The remainder of the preprocessing pipeline differed for fixel‐based versus tensor‐based analyses, as described below.

### 2.4 Fixel‐Based Preprocessing

Fixel‐based preprocessing steps were performed using MRtrix3 commands (Tournier et al. 2019). After eddy current correction, data were converted from NIfTI format to MRtrix's .mif format using the *mrconvert* command, then preprocessed according to the MRtrix fixel‐based workflow (D. A. Raffelt et al. 2017). Images underwent denoising (Cordero‐Grande et al. 2019), unringing (Andersson et al. 2016), and bias field correction (Tustison et al. 2010) and were then upsampled to a voxel resolution of 1 mm^3^. Brain masks were calculated using SynthStrip, a deep learning tool for skull‐stripping brain images (Hoopes et al. 2022).

Fiber orientation distribution (FOD) images were computed using multi‐shell, multi‐tissue (Jeurissen et al. 2014) constrained spherical deconvolution (CSD) (Tournier, Calamante, and Connelly 2007), with group‐averaged response functions for each tissue type (white matter, gray matter, and CSF), and then global intensity normalization was performed to make FOD amplitude comparable across subjects. For group‐level analysis, all FOD images were nonlinearly registered (D. Raffelt et al. 2011) to an unbiased, study‐specific FOD template. The template was created using the *pop_template* command in MRtrix3 (Tournier et al. 2019), which iteratively registered and averaged FOD images from a representative subset of 60 participants, who were randomly stratified by age, sex, education, race, and site using “MatchIt” program in R (Ho et al. 2007).

Next, whole‐brain probabilistic tractography was performed on the FOD template to generate 20 million streamlines that were subsequently filtered to 2 million streamlines using the spherical deconvolution‐informed filtering of tractogram (SIFT) algorithm (R. Smith et al. 2020; R. E. Smith et al. 2013).

To define fixels for the statistical analysis, a template WM fixel mask was segmented from the FOD template. Each FOD was warped into template space and FD, and logFC and FDC were computed. We applied a log‐transform to FC to achieve a more normal distribution. Finally, connectivity‐based fixel enhancement was performed to smooth the data.

### 2.5 Fixel‐Based Statistical Analysis

Statistical analysis was performed using the MRtrix command *fixelfcestats*. To test the association of age with fixel measures, a regression analysis was run separately for each measure (FD, logFC, and FDC) using a general linear model (GLM) controlling for sex, imaging site, years of education, race, and intracranial volume as continuous variables. Sex differences have been previously reported in tensor‐based analyses, but have not been reported in FBAs and aging (Hsu et al. 2008). Therefore, we used sex as a covariate. Site effects have been previously reported to effect DWI measurement accuracy (Zhu et al. 2011). Between the rigorous harmonization protocols and including the site as a covariate, we hope to remove any variance of the site in this analysis. Years of education was used as a marker of socioeconomic status as a covariate, which has been identified previously to affect white matter integrity (Hall et al. 2021). Race was self‐reported and coded using six categories (African American/Black, Asian, Caucasian/White, Native Hawaiian/Pacific Islander, multiracial, or another racial identity). While the effects of race on FBAs have not been reported, race has been implicated in tensor‐based metrics (Fani et al. 2022; Okeke et al. 2023); therefore, we included it as a covariate in all analyses. Intracranial volume has been found to significantly influence tensor‐based measures, suggesting the importance of using it as a covariate (Takao et al. 2011). Statistical inference was performed using connectivity‐based fixel enhancement (D. A. Raffelt et al. 2015), using the 2 million streamlines generated from the study‐specific FOD template. Family‐wise error‐corrected *p* values (FWE *p* value) were then computed for each fixel using nonparametric permutation testing with 10,000 permutations (Nichols and Holmes 2002).

For visualization purposes, streamlined segments that crossed significant fixels were isolated and displayed to represent the significant FBA results in white matter bundles. We applied TractSeg, a convolutional neural network‐based approach that segments 72 white matter tracts in the fields of FOD peaks meant to avoid any bias that may result from user‐defined or atlas‐based delineation (Wasserthal, Neher, and Maier‐Hein 2018; Wasserthal et al. 2019), in order to label the anatomy of each FBA result and identify tracts that contained significant clusters.

To test the associations between fixel measures and cognition, we calculated average values for each FBA metric (FD, logFC, and FDC) across all fixels that were found to be significantly associated with age, separately for fixels that showed a positive or negative correlation with age. Next, we ran GLMs using R studio, examining associations with cognition, controlling for age, sex, site, intracranial volume, education, and race. *p* values for each model were corrected for multiple comparisons using Benjamini and Hochberg's false discovery rate (FDR) *q* level of 0.05, after pooling all *p* values from all models. No missing data were detected for any of the 636 people, and there were no outliers that could be ascribed to poor image quality or inaccuracies.

### 2.6 Tensor‐Based Preprocessing

DTIFIT was performed on each DWI scan after eddy current correction to calculate diffusion parameters. Brain masking was performed using SynthStrip (Hoopes et al. 2022). Tract‐based spatial statistics (TBSS) (Smith et al. 2006) were used to prepare images for analysis. First, nonlinear registration was performed, aligning all FA images to the FMRIB58_FA 1x1x1 standard space. Then, the mean FA image and its derived skeleton, based on FSL's provided TBSS skeleton, was calculated using a threshold of 0.2. AD and MD values were obtained in a similar fashion. Then, using *tbss_non_fa*, he AD and MD maps of each participant were projected onto the mean skeleton.

### 2.7 Tensor‐Based Statistical Analysis

Statistical analyses were performed using the TBSS pipeline in FSL. Unlike FBA, tensor‐based voxel analyses are unable to accommodate crossing fibers and demarcate the boundary between GM and WM, and are therefore highly susceptible to partial volume effects. Pearson partial correlation analyses were performed using a GLM in FSL. The model consisted of the same covariates used in the FBA model, including sex, imaging site, education, race, and intracranial volume. To test the association of age with white matter using voxel measures, a voxel‐wise partial correlation analysis of the TBSS‐derived data and age using a GLM was performed using the permutation analysis of linear models (PALM) framework with 10,000 permutations (Alberton et al. 2020; Winkler et al. 2014) from FSL with 2D threshold‐free cluster‐enhancement (TFCE) (Smith and Nichols 2009) optimization on FA, MD, and AD. In each analysis, correction across both negative and positive contrasts and for multiple voxel‐wise comparisons was performed by controlling for FWE.

### 2.8 Assessing the Performance of FBA vs. TBSS

Model performances were assessed using GLMs with FBA and TBSS metrics and a main effect of age, controlling for sex, imaging site, education, race, and intracranial volume. We extracted the average values from the significant positive and negative regions from FBA and TBSS results separately to examine the equivalency of methods or superiority of one method for detecting associations with age. The Akaike information criterion (AIC) is a metric that is used to compare the fit of several regression models, with a lower AIC indicating better model fit. We also calculated an adjusted *R* ^2^ of each model, which is reflective of the goodness of fit of the model, with higher values indicative of better model fit.

### 2.9 APOE4 Status

Genotypes for the two *APOE* SNPs resulting in six genotypes was performed using TaqMan assays as described previously (Fan et al. 2023). To test if there were white matter differences in APOE4+ (*n* = 180) and APOE4− (*n* = 456), we performed a bivariate linear regression while controlling for all aforementioned covariates.

### 2.10 White Matter Hyperintensities

WMLs were segmented by the Unidentified Bright Object (UBO) detector toolbox (https://cheba.unsw.edu.au/group/neuroimaging‐pipeline; Jiyang et al. 2018) which utilizes SPM12 and FSL functions implemented in Matlab (version 2023b). FLAIR images were registered to each participant's MPRAGE using SPM's co‐registration function (estimate & re‐slice) with Normalized Mutual Information as the objective function. MPRAGE images were segmented into gray matter (GM), white matter (WM), and cerebrospinal fluid (CSF) and the probability maps of the three tissue types were generated. Individual MPRAGE images, co‐registered FLAIR, as well as the GM, WM, and CSF probability maps, were then transformed to DARTEL space, followed by the removal of non‐brain tissue from FLAIR images warped to DARTEL space. Three‐class segmentation (WML, GM/WM, CSF) was conducted on each FLAIR image using FAST for clustering voxels for WML detection. This process considers the spatial intensity variations since WMLs appear bright on FLAIR images. Bias field correction was also conducted on the FLAIR image when calling the FAST function. A supervised learning algorithm, *k*‐nearest neighbors (*k*‐NN), was then used to determine reliable WML clusters from among the candidate clusters. Default settings of *k* = 5 and a probability threshold of 0.7 were used for the WML segmentation in the current study. Results were manually checked for segmentation quality. The resulting WML values were log transformed to reduce skewness.

## 3 Results

The 636 participants were, on average, 69.8 years old (±3.73), female (71%), White (75.7%), with 16.31 years (±2.21) of education. Means and standard deviations of demographic variables ar presented in Table 1. All cognitive domains were associated with age, after accounting for aforementioned covariates. Summary statistics are presented in Table 2.

**TABLE 2:** Linear regression summary statistics of the relationship between age and cognitive domains.

Cognitive domain	Mean, SD, [range]	(95% CI), *p*
Processing speed	0.006, 0.63, [−2.31, 1.67]	(−0.06–0.03), *p* < 0.001
Visuospatial	0.003, 0.57 [−1.79, 1.51]	(−0.05–0.03), *p* < 0.001
Episodic memory	0.007, 0.61 [−2.03, 1.52]	(−0.04–0.02), *p* < 0.001
Working memory	0.006, 0.54 [−1.86, 1.41]	(−0.05, −0.03) *p* < 0.001
Attentional control	0.005, 0.59, [−2.29, 1.51]	(−0.06, −0.04), *p* < 0.001

### 3.1 Associations Between Age and White Matter

**FIGURE 1:**
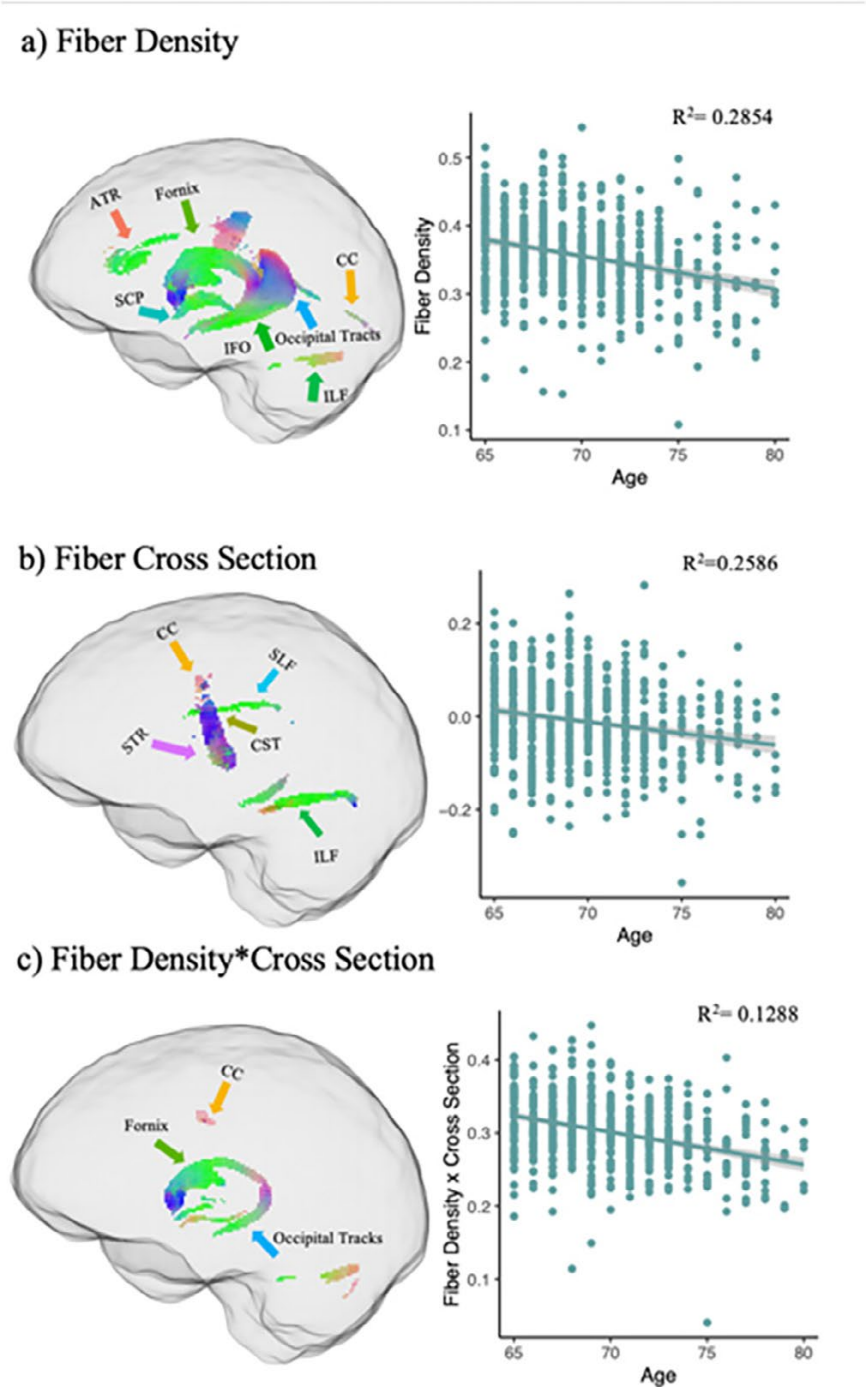
Negative relationships between fixel‐based metrics and age. Fixel‐wise regression analysis between age and baseline fixel‐based metrics, exhibiting significant negative associations with age are shown and color coded by direction (blue = superior–inferior; green = anterior–posterior; red = medial–lateral). (a) FBA showing FD × aging. (b) FBA showing FC × aging. (c) FBA showing FDC × aging. Anterior thalamic radiation (ATR). Corpus callosum (CC), Corticospinal tract (CST), Inferior occipito‐frontal fascicle (IFO), Inferior longitudinal fascicle (ILF), superior cerebellar peduncle (SCP), superior longitudinal fascicle (SLF), superior thalamic radiation (STR).

The TBSS analyses for the traditional tensor‐based approach revealed no significant (FWE *p* < 0.05) associations between age and FA, MD, or AD. Uncorrected maps of the TBSS analysis can be found in the Supporting Information S1: Material 1. In contrast, FBA revealed significant (FWE *p* < 0.05) associations between age and FD, logFC, and FDC (Figure 1) such that increasing age was associated with lower logFC (Figure 1a), FDC (Figure 1b), and FD (Figure 1c). The involved fiber bundles were labeled with TractSeg. See Figure 3 for effect sizes. A full list is in the Supporting Information S1; however, the dominant pathways involved were the fornix (FX) for FD and FDC and the superior thalamic radiation (STR) for logFC. The following tracts were involved in the relationship: arcuate fascicle (AF), anterior thalamic radiation (ATR), commissure anterior (CA), corpus callosum [rostrum (CC 1), genu (CC 2), rostral body (CC 3), anterior midbody (CC 4), posterior midbody (CC 5), isthmus (CC 6), splenium (CC 7)], cingulum (CG), corticospinal tract (CST), middle longitudinal fascicle (MLF), fronto‐pontine tract (FPT), FX, inferior cerebellar peduncle (ICP), inferior occipito‐frontal fascicle (IFO), inferior longitudinal fascicle (ILF), middle cerebellar peduncle (MCP), optic radiation (OR), parieto‐occipital pontine (POPT), superior cerebellar peduncle (SCP), superior longitudinal fascicle I (SLF I), superior longitudinal fascicle II (SLF II), superior longitudinal fascicle III (SLF III), STR, uncinate fascicle (UF), thalamo‐prefrontal (TPREF), thalamo‐premotor (TPREM), thalamo‐precentral (TPREC), thalamo‐postcentral, (T_POSTC), Thalamo‐parietal (T_PAR), thalamo‐occipital (T_OCC), striato‐fronto‐orbital (ST_FO), striato‐prefrontal (ST_PREF), striato‐premotor (ST_PREM), striato‐precentral (ST_PREC), striato‐postcentral (ST_POSTC), striato‐parietal (ST_PAR), striato‐occipital (ST_OCC).

**FIGURE 2:**
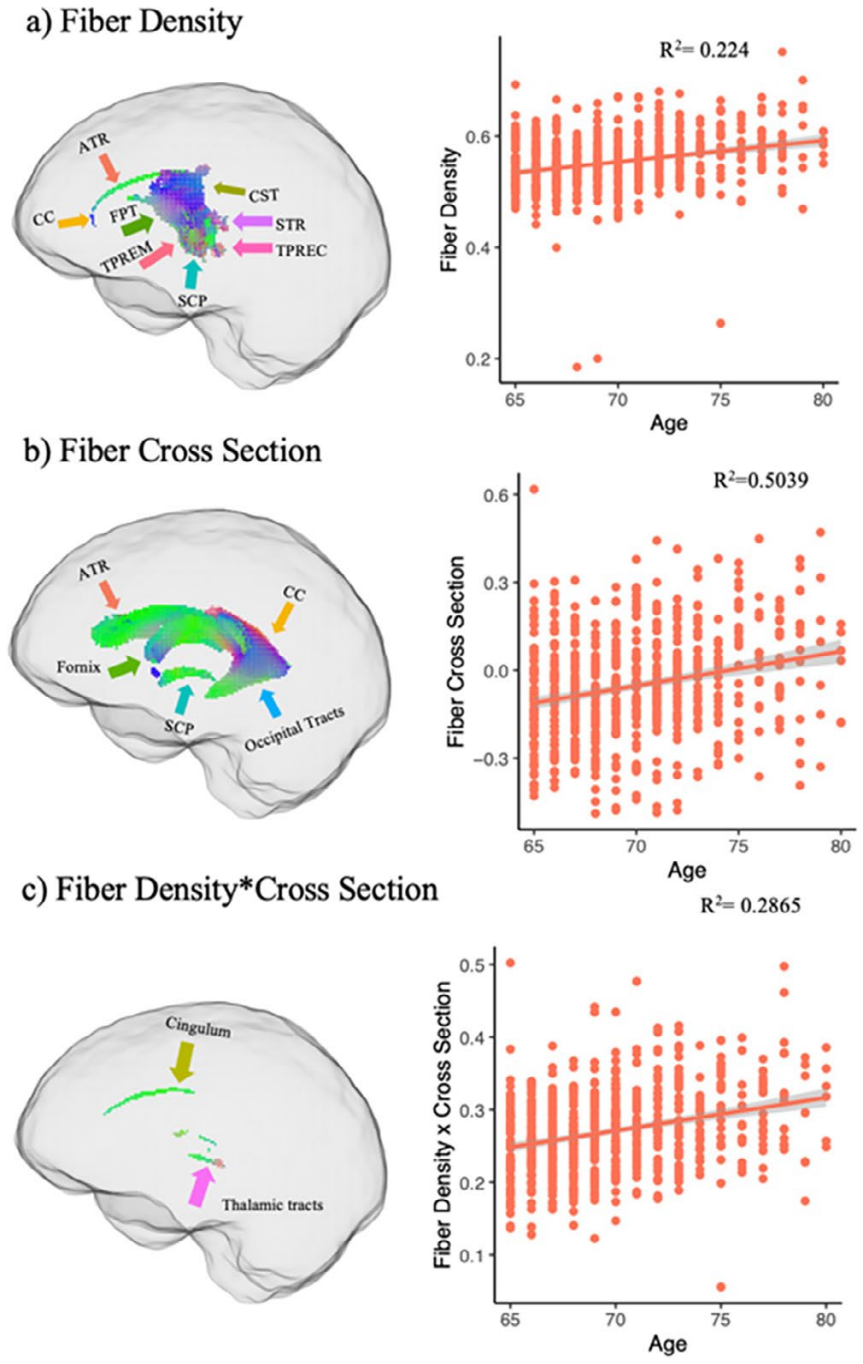
Positive relationship between fixel‐based metrics and age. Fixel‐wise regression analysis between age and baseline fixel‐based metrics, exhibiting significant positive associations with age are shown and color coded by direction (blue = superior–inferior; green = anterior–posterior; red = medial–lateral). (a) FBA showing FD × aging. (b) FBA showing FC × aging. (c) FBA showing FDC × aging. Anterior thalamic radiation (ATR), corpus callosum (CC), corticospinal tract (CST), fronto‐pontine tract (FPT), thalamo‐prefrontal (TPREF), thalamo‐premotor (TPREM), superior cerebellar peduncle (SCP), superior thalamic radiation (STR).

**FIGURE 3:**
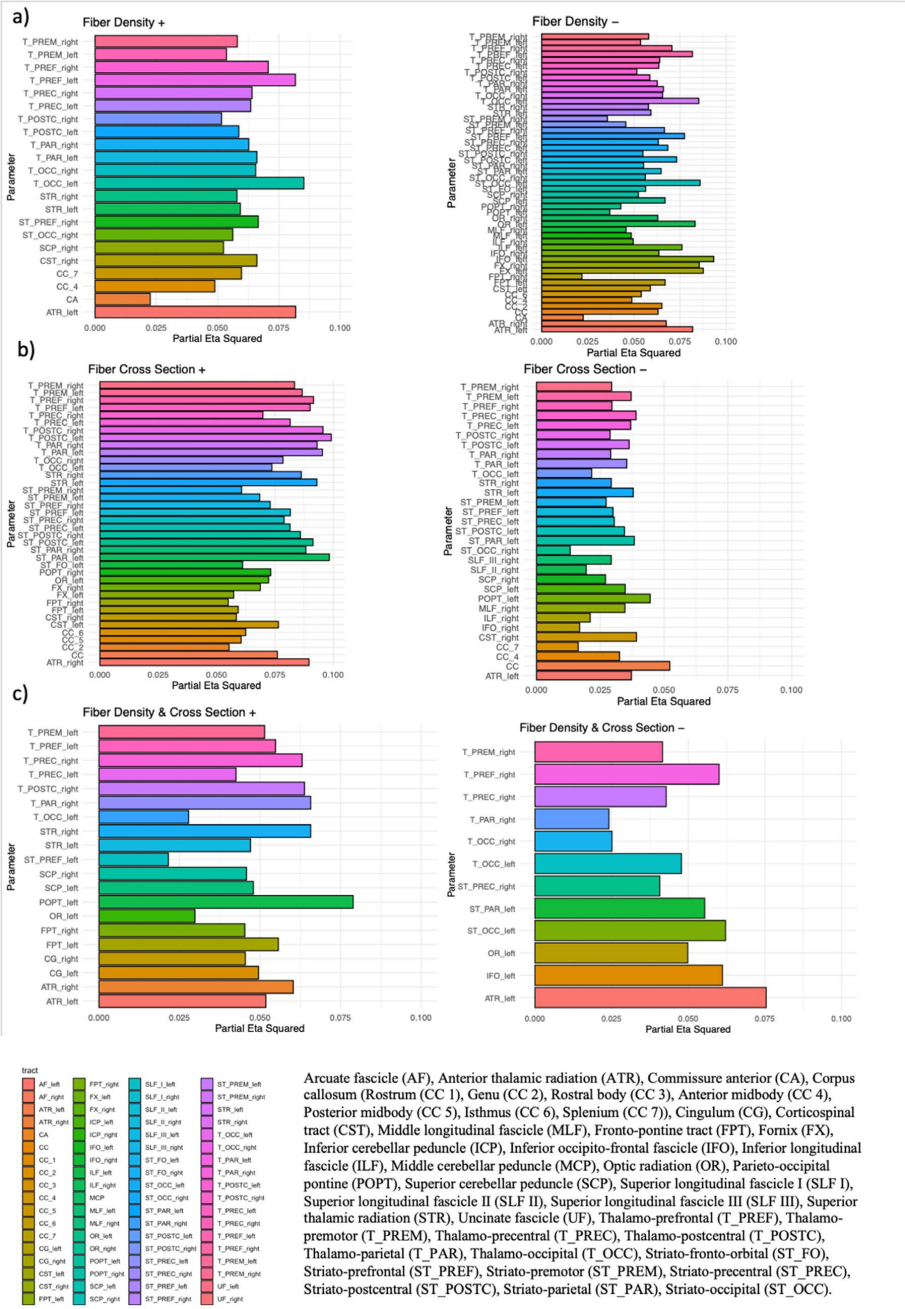
Effect sizes of each metric and associated tract. The color of each bar represents each tract. For FD+, left anterior thalamic radiation and left thalamic occipital tract had the largest effect of age. For FD−, the fornix had the largest effect. For FC+, right thalamo‐postcentral had the largest effect. For FC−, the corpus callosum had the largest effect. For FDC+, the left parieto‐occipital pontine had the largest effect. For FDC−, left anterior thalamic radiation had the largest effect.

FBA revealed significant (FWE *p* < 0.05) positive associations between age and logFC (Figure 2a), FDC (Figure 2b), and FD (Figure 2c) in regions distinct from those that showed decreases with age. The involved fiber bundles were labeled with TractSeg. A full list is in the Supporting Information S1; however, the dominant pathways involved were the fornix for FD, the SCP for logFC, and the cingulum for FDC. See Figure 3 for effect sizes. In contrast, the TBSS analyses revealed no significant (FWE *p* > 0.05) positive associations between age and FA, MD, or AD. Uncorrected maps of the TBSS analysis can be found in the Supporting Information S1.

### 3.2 Assessing the Performance of FBA vs. TBSS

While TBSS did not reveal significant associations with age that passed the FWE threshold, we identified and extracted the average value across voxels that did not pass the FWE threshold, but that reached an uncorrected *p* < 0.05. Similarly, from the FBA approach, we extracted the average value across fixels and voxels for each metric and direction separately that showed a significant association (*p* < 0.05) with age to compare model performance between TBSS pipelines and FBA pipelines. Not surprisingly, all of the fixel‐based measures had superior model fit compared to the tensor‐based measures according to both the AIC and adjusted *R* ^2^ (Table 3). The relationship between FDC and FA in each tract was also tested, to examine whether tensor‐based metrics and fixel‐based metrics were correlated. In each tract, FA and FDC were highly correlated (*r* > 0.34, *p* < 0.01). The tracts with the strongest associations were in the CC and the IFO. See Supporting Information S1 for full summary table.

**TABLE 3:** GLM performance of white matter × age analysis including AIC, adjusted *R* ^2^, and 95% confidence interval of regression coefficient and *p* values.

	AIC	Adjusted *R* ^2^	(95% CI), *p*
FBA metrics			
Fiber density+	2119.627	0.2241	(2.9e−03, 4.8e−03), *p* < 0.001
Fiber cross‐section+	785.6161	0.5039	(6.9e−03, 1.24e−02), *p* < 0.001
Fiber density × cross‐section+	2002.039	0.2865	(3.1e−03, 5.2e−03), *p* < 0.001
Fiber density−	1958.592	0.2854	(−5.3e−03, −3.1e−03), *p* < 0.001
Fiber cross‐section−	1450.777	0.2586	(−7.4e−03, −4.2e−03), *p* < 0.001
Fiber density × cross‐section−	2161.865	0.1288	(−5.2e−03, −3.3e−03), *p* < 0.001
TBSS metrics			
Mean diffusivity+	12,306.14	0.0882	(7.4e−07, 1.4e−06), *p* < 0.001
Axial diffusivity+	11,740.96	0.1384	(6.7e−07, 1.7e−06), *p* < 0.001
Fractional anisotropy+	3352.703	0.1668	(−1.6e−07, 5.6e−07), *p* = 0.58
Mean diffusivity−	12,140.64	0.0166	(−1.6e−07, 5.6e−07), *p* = 0.27
Axial diffusivity−	11,762.11	0.0262	(−4.5e−07, 5.2e−07), *p* = 0.89
Fractional anisotropy−	3100.632	0.1182	(−1.9e−03, −1.0e−03), *p* < 0.001

*Note:* + indicates metrics extracted from the positive relationship between age and white matter while − indicates metrics extracted from the negative relationship between age and white matter.

### 3.3 Tract‐Based Results

To gain a better understanding of the tract specificity of these results, FDC, FC, and FDC were calculated per each tract. Then, associations were examined between per tract FDC, FC, and FD and age. A summary of the results are presented in Table S3. For FDC, CC_2, CC_4, CC_6, CC, FX_left, FX_right, IFO_right, MLF_right, OR_left, OR_right, SLF_III_right, ST_OCC_left, ST_OCC_right, ST_PAR_right, ST_POSTC_left, T_OCC_left, T_OCC_right, T_PAR_right were all significantly negatively associated with age such that a decrease in FDC in this region was associated with increasing age. For FD, CA, CC_4, CC_5, CC_6, CC_7, CC. CST_left, CST_right FPT_left, FPT_right, FX_left, FX_right, IFO_right, MLF_left, MLF_right, SCP_left, SCP_right, SFL_III_left, ST_FO_left, ST_PAR_left, ST_POSTC_left, ST_POSTC_right, ST_PREC_right, T_OCC_right, T_PREM_right, UF_left were all significantly negatively associated with age such that a decrease in FD in this region was associated with increasing age. For FC, ATR_right, ATR_left, FX_right, IFO_right, MLF_left, MLF_right, OR_left, OR_right, POPT_right, SCR_right, SLF_III_left ST_OCC_left, ST_OCC_right, ST_POSTC_left, ST_POSTC_right, ST_PREC_right, ST_PREC_left, ST_PREF_left, ST_PREF_right, T_OCC_left, T_OCC_right, T_PREC_right, T_PREM_left, and UF_right were all significantly negatively associated with age such that a decrease in FC in this region was associated with increasing age. Overall, the results were widespread, however, the associations with age on these metrics slightly favored the right‐sided tracts more than the left‐sided tracts. Out of 24 tracts that demonstrated a relationship between FC and age, 13 of them were right‐sided. Out of the 20 bilateral tracts for FD, 10 tracts were right sided. Out of the 13 bilateral tracts for FDC, 9 tracts were right‐sided. See Supporting Information S1 for full results and table.

### 3.4 White Matter and Cognitive Performance

**FIGURE 4:**
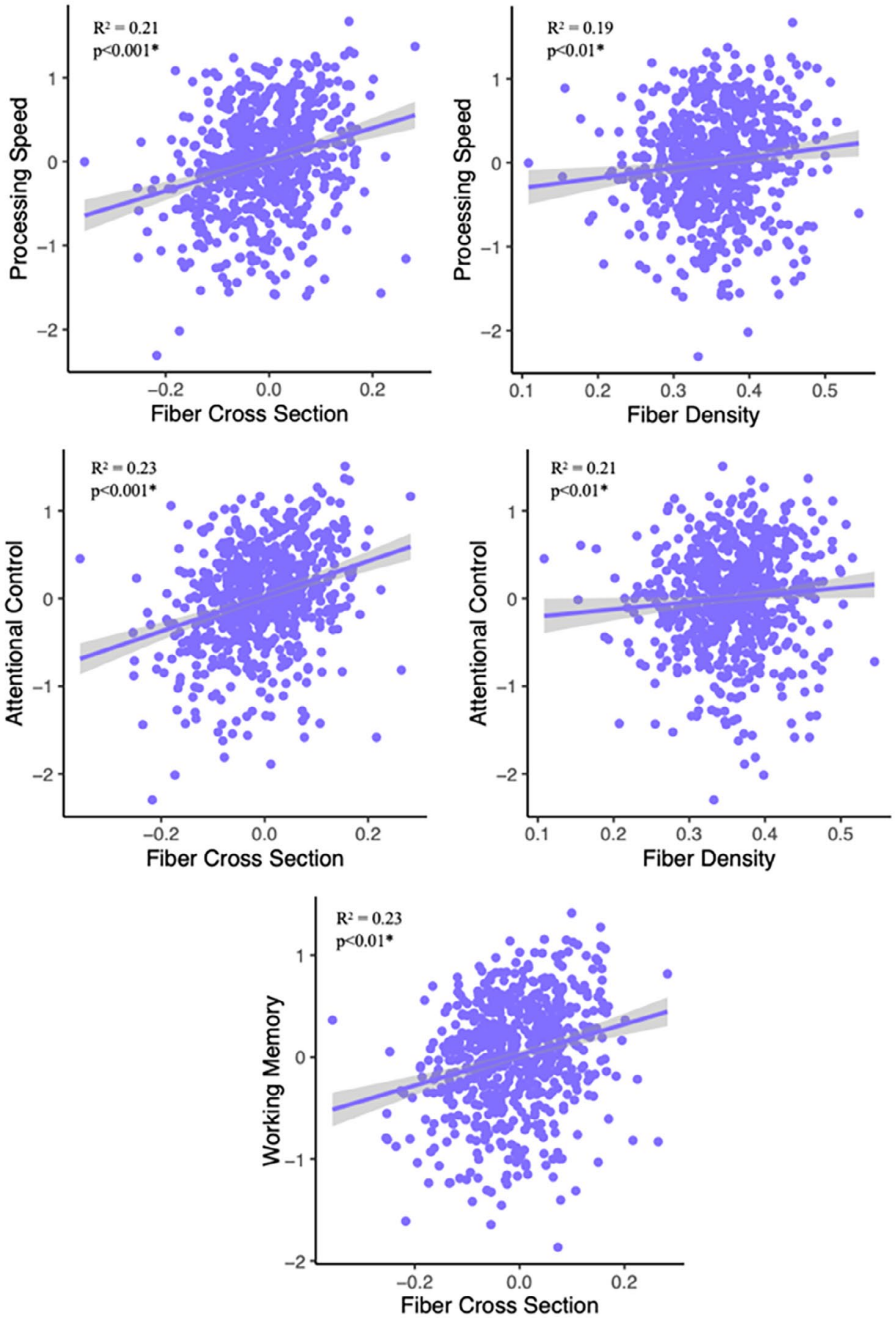
Regression graphs demonstrating the significant positive relationships with age‐related FC and FD and processing speed, attentional control, and working memory.

Examining the associations between FBA metrics (from the fixels negatively associated with age) and cognitive performance revealed that FD and FC were both significantly (FDR *p* < 0.01) associated with processing speed [FD: *β* = 1.36, 95% CI (0.04, 0.20), *p* < 0.01] [FC: *β* = 1.27, 95% CI (0.09, 0.20), *p* < 0.001], and attentional control [FD: *β* = 1.22, 95% CI (0.04, 0.20), *p* < 0.01] [FC: *β* = 0.87, 95% CI (0.08, 0.20), *p* < 0.001], and working memory was associated with FC (*β* = 0.87, 95% CI (0.38, 1.35), *p* < 0.001) (Figure 4), such that lower FBA values were associated with poorer performance in these cognitive domains. Associations between cognitive performance and FBA results is presented in Table 4. There were no significant associations between cognitive performance and values from the FBA clusters that were positively associated with age (Supporting Information S1).

**TABLE 4:** FBA × cognitive results from GLM.

	Fiber density	Fiber cross‐section	Fiber density × cross‐section
Processing speed	*β* = 1.36, *p* < 0.01*	*β* = 1.44, *p* < 0.001*	*β* = 0.11, *p* = 0.08
Visuospatial	*β* = 0.62, *p* = 0.23	*β* = 0.63, *p* = 0.06	*β* = 0.12, *p* = 0.85
Episodic memory	*β* = −0.18, *p* = 0.85	*β* = −0.17, *p* = 0.68	*β* = −0.64, *p* = 0.42
Working memory	*β* = 0.81, *p* = 0.07	*β* = 0.87, *p* < 0.001*	*β* = 0.29, *p* = 0.64
Attentional control	*β* = 1.24, *p* < 0.01*	*β* = 1.36, *p* < 0.001*	*β* = 0.90, *p* = 0.09

* All *p* values in this table are FDR corrected.

**FIGURE 5:**
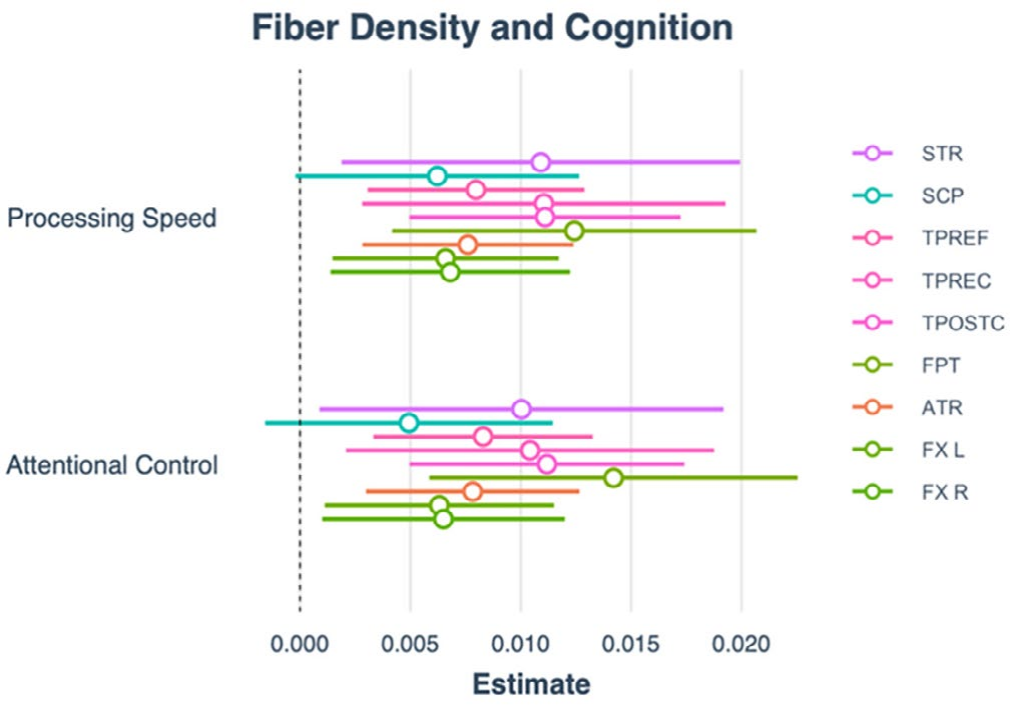
Plot demonstrating beta coefficients and confidence intervals for each tract and processing speed and attentional control.

To identify if there were regional differences in the strength of these associations, we identified all tracts (output from TractSeg) where > 5% of the fixels within each tract showed a significant negative correlation with age (Supporting Information S1). This threshold was designed to focus the analysis on tracts showing the strongest degree of involvement. These tracts were the left superior thalamic radiation (STR), left superior cerebellar peduncle (SCP), left thalamo‐precentral (TPREC), left thalamo‐postcentral (TPOSTC), left thalamo‐prefrontal (TPREF), left fronto‐pontine tract (FPT), fornix (FX), and left ATR and only in FD. FTP was the tract most involved in processing speed and attentional control. For processing speed, the SCP was the least involved. For attentional control, the STR was the least involved (Figure 5; Table 5).

**TABLE 5:** Linear regression results demonstrating the relationship between significant tracts associated with age and processing speed and attentional control. Fully adjusted *R* ^2^ and FDR‐corrected *p* values are presented.

	FD superior thalamic radiation (L)	FD superior cerebellar peduncle (L)	FD thalamo‐prefrontal (L)	FD thalamo‐precentral (L)	FD thalamo‐postcentral (L)	FD fronto‐pontine tract (L)	FD anterior thalamic radiation (L)	FD fornix (L)	FD fornix (R)
Processing speed	*R* ^2^ = 0.12 *p* = 0.02	*R* ^2^ = 0.23 *p* = 0.06	*R* ^2^ = 0.27 *p* = 0.004	*R* ^2^ = 0.15 *p* = 0.02	*R* ^2^ = 0.20 *p* = 0.003	*R* ^2^ = 0.19 *p* = 0.003	*R* ^2^ = 0.27 *p* = 0.005	*R* ^2^ = 0.19 *p* = 0.02	*R* ^2^ = 0.18 *p* = 0.02
Attentional control	*R* ^2^ = 0.12 *p* = 0.03	*R* ^2^ = 0.23 *p* = 0.14	*R* ^2^ = 0.27 *p* = 0.003	*R* ^2^ = 0.15 *p* = 0.02	*R* ^2^ = 0.20 *p* = 0.003	*R* ^2^ = 0.19 *p* = 0.003	*R* ^2^ = 0.27 *p* = 0.005	*R* ^2^ = 0.19 *p* = 0.02	*R* ^2^ = 0.19 *p* = 0.02

## 4 Examination of Moderators

### 4.1 APOE4 Status

To test if there were white matter differences in APOE4+ (*n* = 180) and APOE4− (*n* = 456), we performed a bivariate linear regression while controlling for all aforementioned covariates. Results showed no differences between APOE4 status and FD, logFC, and FDC (FDR *p* > 0.05) (Table 6).

**TABLE 6:** GLM performance of white matter × age analysis with WMH as a covariate including beta estimates, adjusted *R* ^2^, and 95% confidence interval and *p* values.

	APOE4+ (mean)	APOE4− (mean)	(95% CI), FDR *p*
Fiber density−	0.358541	0.3549122	(−0.07, 0.07), 0.99
Fiber cross‐section−	−0.005633694	−0.01279827	(−0.02, 0.07), 0.302
Fiber density × cross‐section−	0.303	0.301	(−0.09, 0.08), 0.98
Fiber density+	0.5476115	0.5552869	(−1.23, 0.32), 0.25
Fiber cross‐section+	−0.069566	−0.04824326	(−0.41, 0.14), 0.33
Fiber density × cross‐section+	0.2724315	0.2631334	(−1.2, 0.21), 0.17

### 4.2 White Matter Hyperintensities

To test if there was any effect of WMH on the associations between fixel metrics and age or fixel metrics and cognition, we added WMH load as a covariate to the linear regressions performed. No significant change was seen to the results, suggesting that WMH load was not influencing the relationship between fixel metrics and age (Table 7), or fixel metrics and cognition (Table 8).

**TABLE 7:** GLM performance of white matter × age analysis with WMH as a covariate including beta estimates, adjusted *R* ^2^, and 95% confidence interval and *p* values.

	*β*	Adjusted *R* ^2^	(95% CI), *p*
Fiber density+	3.615e−03	0.2374	(2.7e−03, 4.6e−02), *p* < 0.001
Fiber cross‐section+	8.825e−03	0.499	(5.9e−03, 1.2e−02), *p* < 0.001
Fiber density × cross‐section+	4.264e−03	0.2796	(3.2e−03, 5.3e−03), *p* < 0.001
Fiber density−	−3.770e−03	0.3072	(−4.8e−03, −2.7e−03), *p* < 0.001
Fiber cross‐section−	−4.792e−03	0.2949	(−6.4e−03, −3.2e−03), *p* < 0.001
Fiber density × cross‐section−	−3.905e−03	0.1549	(−4.8e−03, −2.9e−03), *p* < 0.001

**TABLE 8:** FBA × cognitive results from GLM with WMH as a covariate.

	Fiber density	Fiber cross‐section	Fiber density × cross‐section
Processing speed	*p* = 0.01*	*p* < 0.001*	*p* = 0.09
Visuospatial	*p* = 0.40	*p* = 0.15	*p* = 0.88
Episodic memory	*p* = 0.59	*p* = 0.52	*p* = 0.29
Working memory	*p* = 0.08	*p* = 0.14	*p* = 0.71
Attentional control	*p* = 0.01*	*p* < 0.001*	*p* = 0.14

* All *p* values are FDR corrected (FDR *p* < 0.05).

### 4.3 Sex Differences

Bivariate linear regressions showed no sex differences between FD−, log FC−, FDC−, log FC+, and FDC+ (FDR *p* > 0.05), but did show differences in logFC+ (Table 9).

**TABLE 9:** Sex differences in FBA metrics.

	Female (mean)	Male (mean)	(95% CI), FDR *p*
Fiber density−	0.3675065	0.3282518	(−0.02, 0.09), 0.19
Fiber cross‐section−	−0.02689438	0.03048495	(−0.08, −0.005), 0.06
Fiber density × cross‐section−	0.3049647	0.2946148	(0.003, 1.34), 0.07
Fiber density+	0.5503606	0.560409	(−0.009, 0.36), 0.25
Fiber cross‐section+	−0.09670063	0.05083859	(0.87, 2.14), 0.001*
Fiber density × cross‐section+	0.259867	0.296724	(−0.61, 0.57), 0.95

*FDR *p* < 0.05.

**FIGURE 6:**
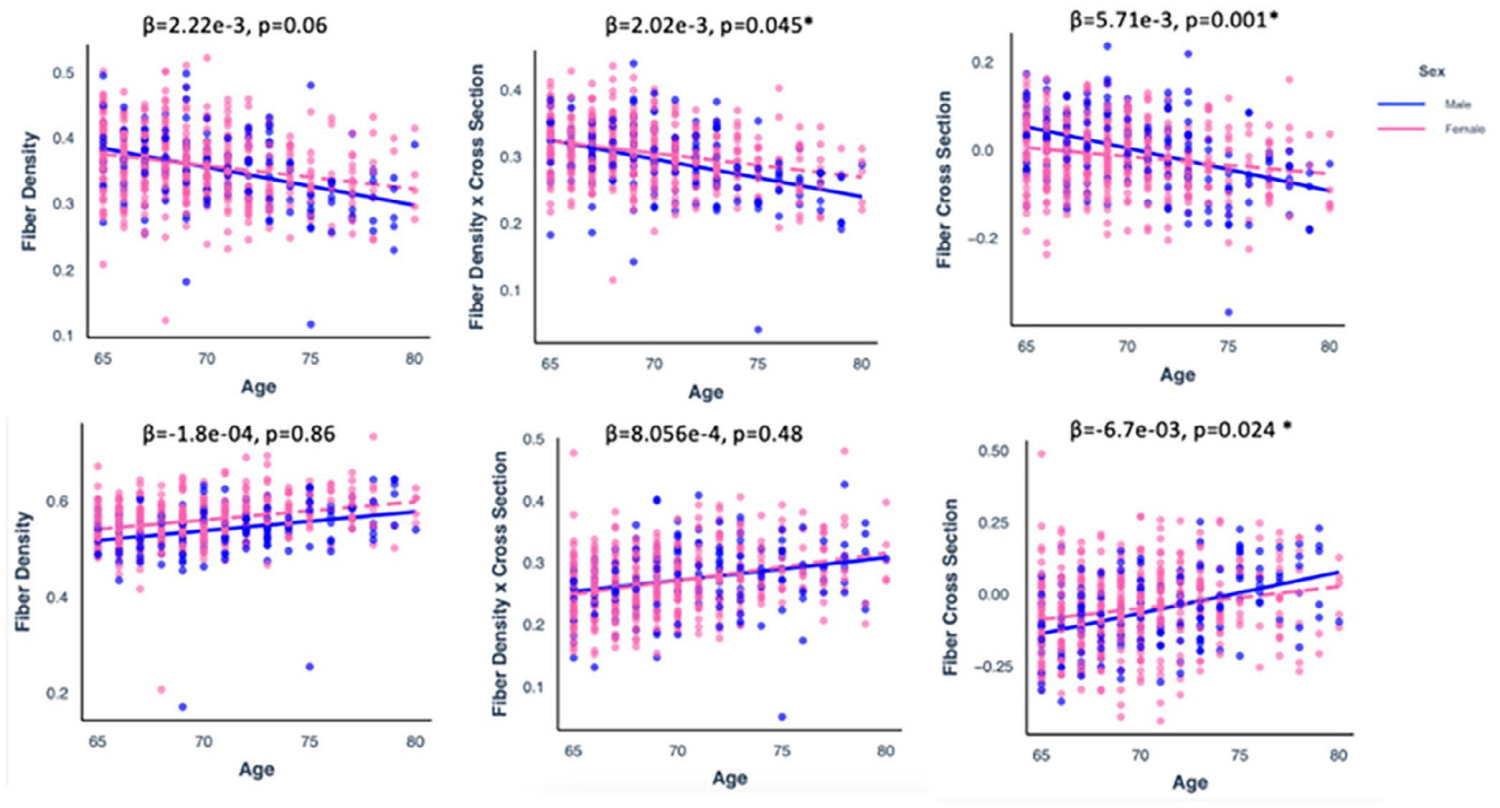
Scatter plots showing a significant positive association between age (*x*‐axis) and FBA metric (on the *y*‐axis) in males (blue) and females (pink).

Separate GLMs, including an age × sex interaction term, were performed with FD, log FC, and FDC from the positive and negative age‐related regions (Figure 6). A significant *age* × *sex* interaction was found for FDC− [*β* = 2.02e−3, SE = 1.01e−03, 95% CI (4.67e−05, 3.99e+05), *p =* 0.62], logFC− [*β* = 5.71e−3, SE = 1.18e−03, 95% CI (2.27e−03, 9.161e−03), *p =* 0.001], logFC+ [*β* = −6.7e−03, SE = 2.97e−03, 95% CI (−1.3e−02, −8.7e−04), *p =* 0.024], but not for FD− [*β* = 2.22e−3, SE = 1.043e−03, 95% CI (−1.01e−04, 4.53e−03), *p* = 0.06], FDC+ [*β* = 8.056e−4, SE = 1.143e−03, 95% CI (−1.43e−03, 3.05e−03), *p* = 0.48], or FD+ [*β* = −1.8e−04, SE = 1.043e−03, 95% CI (−1.25e−02, −8.69e−04), *p* = 0.86]. Post‐estimation FDR‐corrected simple slopes showed there was a significant negative association between age and all fixel‐based metrics (FD, logFC, and FDC) in males (*p* < 0.001) and females (p < 0.001); however, the strength of association was stronger in males than in females (Figure 5). This suggests a greater decline in white matter integrity with greater age in males compared to females.

## 5 Discussion

Consistent with our predictions, we found that FBA metrics (FD, logFC, FDC) were significantly associated with age and cognition. The results support our hypothesis that FBA is more sensitive to age‐related white matter differences in older adults than traditional approaches that are unable to decompose crossing fibers. Furthermore, these findings indicate that age‐related differences in white matter integrity may be expressed differently across the brain, with some pathways showing increases with advancing age and other regions showing decreases. However, only the regions negatively associated with age were linked with cognitive performance.

It is interesting that the tensor‐based analysis did not show significant associations with age. Prior research on diffusion‐weighted MRI has consistently found associations between age and tensor‐based measures, which raises the question of why we failed to show similar patterns in the current sample. One reason might be because of the methods used to correct for multiple comparisons. The current analysis utilized FWE, which could be more conservative than other statistical thresholding approaches that have been utilized for tensor‐based analyses. In support of this argument, the uncorrected tensor‐based maps, such as FA (Supporting Information S1), showed subthreshold associations with age. In contrast, the FBA approach also used an FWE‐corrected model, and found significant results, highlighting the more robust capabilities of FBA. Another possible explanation for our failure to find age associations with traditional approaches is that our study had a relatively restricted age range, whereas most prior work examining white matter microstructural differences were conducted on samples with broader age ranges (e.g., across the adult lifespan) (Beck et al. 2021; Cox et al. 2016; Sexton et al. 2014; Westlye et al. 2010; Yap et al. 2013). A potential reason for fixel‐based measures being more sensitive may be due to the ability of FBA to detect fiber‐specific populations within each voxel, thus addressing the challenges of interpreting the impact of crossing fibers in tensor‐based approaches (Raffelt et al. 2017). Overall, these findings support the hypothesis that fixel‐based measures have greater sensitivity for detecting age‐related white matter differences compared to tensor‐based metrics, even in a constricted age range (Han et al. 2023; Kelley et al. 2021; Sakamoto et al. 2021).

Combining both tensor and fixel‐based metrics and examining how these metrics present similarly in different tracts may provide us with a better understanding of the biological underpinning of the association with age on these tracts. After calculating the correlation coefficient for the relationship between FA and FDC for each tract, the CC and the IFO had the strongest association (*r* > 0.6, *p* < 0.001). This suggests that there is both microstructural and macrostructural damage happening in these tracts as a function of age and that they share some elements but are also not redundant. Thus, it is the unique method of FDC and what it captures that is driving the association with age and cognition. Prior work has also shown associations between FA in the CC and IFO and aging (Sala et al. 2012). In addition, the association with age and the CC and IFO has been shown in prior work (Kelley et al. 2021). This association between FA and FDC may also reflect dimensional changes in FDC and variability across fiber populations within a voxel (Douaud et al. 2011; Kelley et al. 2021). Although our model fit indices indicate superior model fit for the FBA, this shouldn't be a surprise since the values extracted indicated significant versus nonsignificant associations with age. Thus, the interpretations of these models should be interpreted with caution.

Increasing age was significantly associated with a decline in all three fixel‐based measures of white matter fiber integrity. Our results found widespread associations between decreasing FDC with increasing age, replicating prior work (Han et al. 2023; Kelley et al. 2021; Zivari Adab et al. 2020). As the product of FD and FC, FDC is a measure of overall changes to the microstructure of the fiber. Thus, this negative relationship with age may reflect the loss of myelinated fibers and the degeneration of white matter fiber bundles (Marner et al. 2003; Peters 2009).

Increasing age was also significantly associated with greater FD, FC, and FDC. Particularly for FD and FDC, these associations surrounding the periventricular regions, suggest that this could be mediated by increased neuroinflammation. Compensatory neuroplasticity in response to neurodegeneration is another plausible explanation. Prior studies have also discovered counter‐intuitive findings, such as increased cortical thickness (Phan et al. 2024) and mixed results in diffusion metrics (Kuchtova et al. 2018; Wurst et al. 2023) in Alzheimer's disease patients. These prior studies suggest that asymmetrical compensatory changes under conditions of neuropathology may be redirecting and utilizing other structures. Another interpretation may be that increases in FC and FD are related to a heightened state of neuroinflammation and increased extra‐axonal space filling with extracellular materials.

**FIGURE 7:**
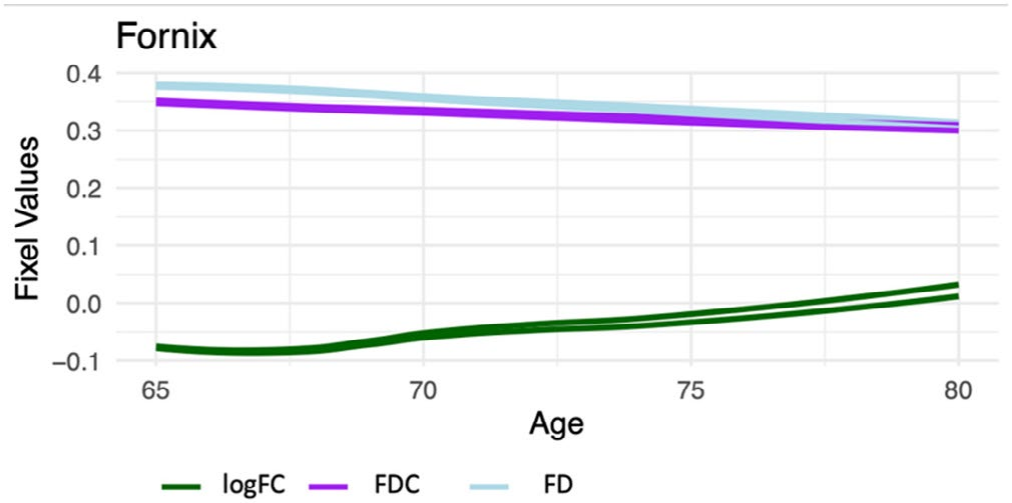
Line graph demonstrating the relationship between FD, FC, and FDC in the FX (right and left) with age.

Interestingly, the regions that showed a negative correlation with FD also showed positive correlations with FC. This replicates several prior studies (Choy et al. 2020; Han et al. 2023) and suggests that these regions, namely the FX, thalamic, and striatal tracts, have decreased packing of fibers together with an increase in fiber bundle cross‐section. Opposingly, regions that were positively associated with age in FD were negatively associated with age in FC, suggesting that these regions have an increased packing of fibers rather than an enlarged fiber diameter. There are several possible explanations for why some brain metrics decrease and some increase in various populations. Specifically, the FX seems to play a large role in these changes (Figure 7, see Supporting Information S1 for graphs of all tracts). When both axonal loss and atrophy occur, a complex set of effects play out, with FD increasing and FC decreasing (Dhollander et al. 2021). For example, the cingulate bundle of aged monkeys showed less tightly packed axonal myelinated nerve fibers, indicating fiber loss, as well as ballooning of the sheaths (Bowley et al. 2010). Other underlying neural substrates, such as lesion‐induced axonal loss, gliosis, and disrupted macrostructural organization, may also be responsible for these mixed results (Bennett et al. 2010). Importantly, in our study, the regions showing positive associations with age were unrelated to cognitive performance. Therefore, the behavioral implications of these associations remain a matter of speculation.

There are several regions that had large magnitude associations with age in this analysis, with the greatest being the FX. Almost half of the FX showed that increasing age was associated with higher FC and lower FDC (Figure 4). Interestingly, prior work has shown that the FX is involved in memory consolidation (Benear, Ngo, and Olson 2020) and decreases with age (Korbmacher et al. 2023). Atrophy is seen in the FX both in MCI (Fletcher et al. 2013) and Alzheimer's disease (Mielke et al. 2012). Moreover, new research has shown that the FX is a potential target for deep brain stimulation, modulating associative and limbic networks that serve memory functions (Aldehri et al. 2018). Furthermore, precise regions of the FX, such as the circuit of Papez and stria terminalis, have become targets for DBS to improve cognition in Alzheimer's disease (Ríos et al. 2022). In our analysis, the FX was the tract with the most consistent age‐related degeneration (Supporting Information S1). This warrants further investigation into interventions targeting degenerative processes happening in the FX. There are several other tracts that were associated with age in this analysis, namely the striatal and thalamic tracts. These tracts show the largest effect sizes in the FC+ and FD− analyses (Supporting Information S1), although each significant region was only a small portion of the entire tract.

While results indicated that there were tracts both positively and negatively associated with increasing age, only the regions that were negatively associated with age were also related to cognitive performance. Specifically, only FC and FD were related to processing speed and attentional control. Reduced processing speed is a common feature of cognitive aging and has been discussed as a common cause of other cognitive deficits. White matter degeneration may contribute to reduced processing speed in healthy aging by affecting information transfer speed (Madden et al. 2012; O'Sullivan et al. 2001; Voineskos et al. 2012). In addition, both FD and FC were also associated with attentional control, a subcomponent of executive function. Prior work has shown that tracts connecting frontal brain regions with thalamic projections and with the parietal lobes are most associated with age‐related decline on attentional control tasks (Grieve et al. 2007; Jolly et al. 2017; Li et al. 2018). Our results indicate that both processing speed and attentional control are related to decreasing fiber bundle size and density in older adults.

It is important to note the presence of genetic factors that may influence cognitive aging and white matter degeneration. Prior work has demonstrated polymorphisms of APOE4 represent genetic risk factors for dementia and cognitive impairment in older adults. Moreover, previous research has shown a decline in white matter integrity in APOE4 carriers (Operto et al. 2018; Persson et al. 2006). To tease out if white matter integrity, assessed by FBA, was related to APOE4 status, we tested the association between APOE4 status and fixel‐based metrics and found no significant difference between APOE4+ and APOE4− (Supporting Information S1: Material 5). These results suggest that this decline in white matter integrity with age is unrelated to APOE4 carriage in older adults.

WMH are commonly seen in older adults, resulting from chronic ischemia and representing myelin and axonal loss (Prins and Scheltens 2015). Cognitive aging is also affected by WMH, particularly impairing information processing speed, leading to executive dysfunction (Prins and Scheltens 2015). We performed an exploratory analysis to determine if adding WMH load as a covariate changed the results. The patterns described above were unchanged after adding WMH as a covariate, suggesting that WMH were not affecting the fixel‐based results.

The main limitation of this study is that our results are cross‐sectional. Thus, we cannot comment on longitudinal changes in white matter over the course of aging. In addition, participants were low‐active and voluntarily signed up for a 1‐year exercise intervention. Another limitation is that our sample comprised cognitively unimpaired participants. However, our range of cognitive performance was still broad enough to detect variations in other markers of health and function. Future longitudinal studies should assess how white matter declines with age and its association with cognitive decline and transition into cognitive impairment.

There are many strengths to this study. The population is a large and well‐characterized sample. Moreover, we leveraged a cutting‐edge analytical approach, comparing it to traditional analysis approaches. Furthermore, we used a comprehensive cognitive battery including multiple domains of cognition. This study warrants future work to address other factors that may contribute to accelerated brain aging, such as cardiovascular risk (España‐Irla et al. 2021), medications (Risacher et al. 2016), sleep (Scullin 2017), diet (Pelletier et al. 2015), exercise (Erickson, Gildengers, and Butters 2013; Erickson, Hillman et al. 2019), fitness (Colcombe et al. 2003, 2006; Polk et al. 2023; Voss et al. 2013), and sedentary time (Ai et al. 2024; Maasakkers et al. 2022; Morris et al. 2022).

## 6 Conclusions

This study investigated the relationship between fixel‐based metrics and age in a cognitively unimpaired, older adult sample. Our results indicate that fixel‐based metrics are more sensitive to the aging of white matter relative to tensor‐based metrics. Exploratory analyses revealed that APOE4 status and WMH did not moderate the relationship between age and fixel‐based metrics, but sex did. In addition, our results indicate that white matter decline in regions that are associated with aging are also associated with cognitive performance. This study provides a critical framework for future analyses utilizing FBA in older adults. Overall, these findings demonstrate proof of concept, sensitivity, and utility for using FBA to detect age‐related white matter decline and its relationship to cognitive performance in a restricted age range of older adults.

## Conflicts of Interest

The authors declare no conflicts of interest.

## Supporting information


**Data S1.** Supporting Information.

## Data Availability

Data will be made available upon request. Code for preprocessing and analyses are available freely at https://github.com/emmatinney/ignite_fba.
