# Supplementary material for: Understanding Cognitive Aging Through White Matter: A Fixel‐Based Analysis
Source: Hum Brain Mapp. 2024 Dec 25;45(18):e70121. doi: 10.1002/hbm.70121 (PMC11669003; doi:10.1002/hbm.70121)
Supplement: Supplementary file 1 — Data S1. Supporting Information. [file HBM-45-e70121-s001.docx]

Supplemental Materials


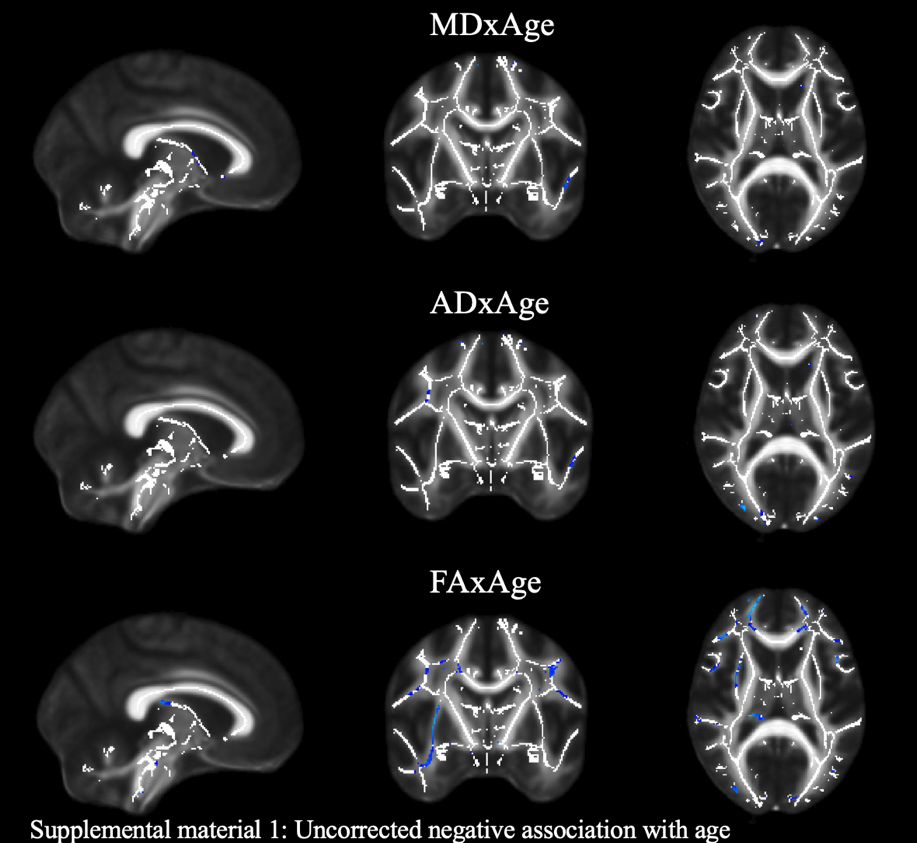


Supplemental Material 1: Significant voxels from MD/AD/FA with significant voxels in blue that show a negative relationship with age thresholded at uncorrected p<0.05.


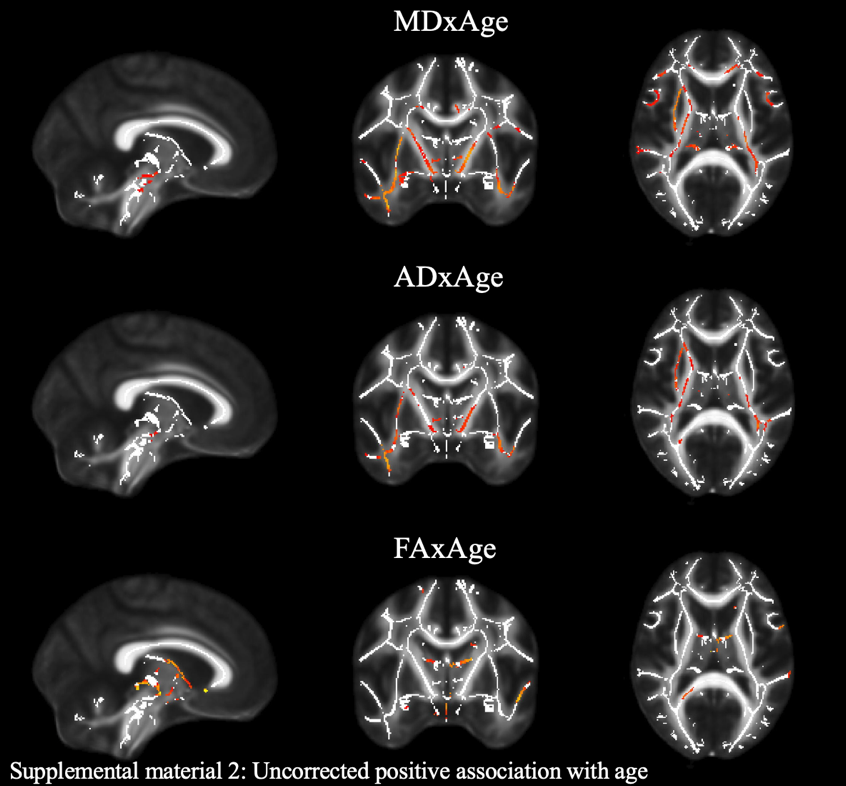


Supplemental Material 2: Significant voxels from MD/AD/FA with significant voxels in red that show a positive relationship with age thresholded at uncorrected p<0.05.

Supplemental Material 3: Regions Negatively associated with age. Table of all tracts normed to size of tract (% of tract with significant streamlines).


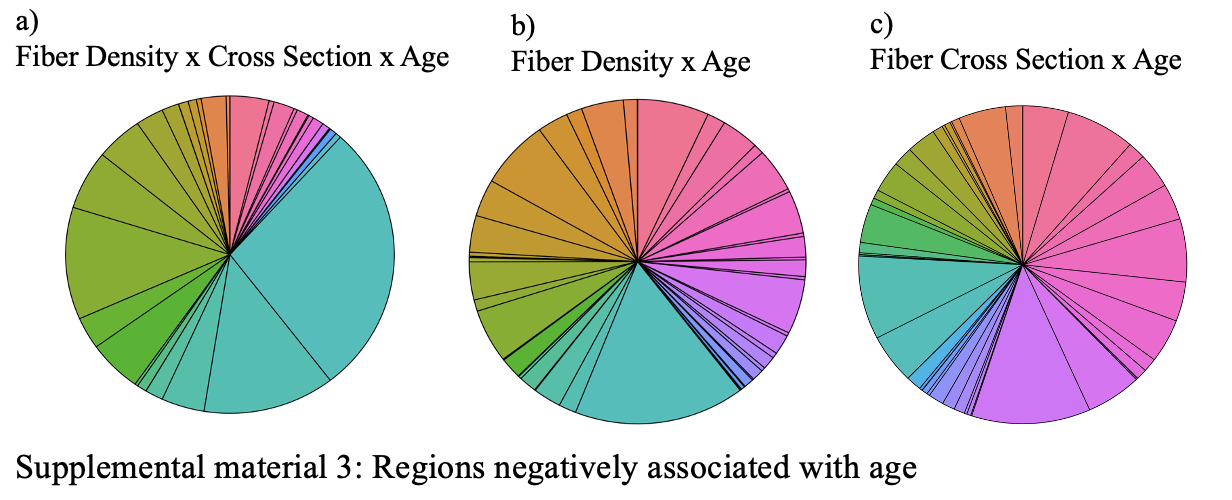


| Tracts | FD x Age (% of tract) | FDC x Age (% of tract) | FC x Age (% of tract) |
| --- | --- | --- | --- |
| AF_left | 0 | 0 | 0 |
| AF_right | 0 | 0.07753245 | 0.65228384 |
| ATR_left | 2.49285673 | 0.22741851 | 1.79602309 |
| ATR_right | 1.36530447 | 0.09330099 | 0.31411333 |
| CA | 0.09082652 | 0 | 0 |
| CC_1 | 0 | 0 | 0 |
| CC_2 | 0.08208471 | 0 | 0 |
| CC_3 | 0 | 0 | 0 |
| CC_4 | 4.28934548 | 0.36274094 | 0.22770599 |
| CC_5 | 3.86882081 | 0 | 0 |
| CC_6 | 1.12218681 | 0.00088292 | 0.10594997 |
| CC_7 | 1.94239523 | 0 | 0.39240308 |
| CC | 1.26318564 | 0.05867372 | 0.06887784 |
| CG_left | 0 | 0 | 0 |
| CG_right | 0 | 0 | 0 |
| CST_left | 0.59804203 | 0 | 1.12235284 |
| CST_right | 0.21176471 | 0.37647059 | 0.75764706 |
| FPT_left | 0.31516313 | 0 | 1.22283294 |
| FPT_right | 0.00925241 | 0 | 0.34542314 |
| FX_left | 83.401222 | 43.4826884 | 0 |
| FX_right | 92.1092564 | 44.9924127 | 0 |
| ICP_left | 0 | 0 | 0 |
| ICP_right | 0 | 0 | 0 |
| IFO_left | 1.74937139 | 0.09670972 | 0 |
| IFO_right | 1.54627886 | 0 | 0.27158439 |
| ILF_left | 1.29503468 | 0 | 0 |
| ILF_right | 0.27236663 | 0 | 1.5157795 |
| MCP | 0 | 0 | 0 |
| MLF_left | 0.85532936 | 0 | 0 |
| MLF_right | 0.59240259 | 0 | 0.4010423 |
| OR_left | 2.52132943 | 0.02971264 | 0.07215926 |
| OR_right | 2.50113459 | 0.02017044 | 0 |
| POPT_left | 0.17221708 | 0 | 0.01291628 |
| POPT_right | 0.40131339 | 0 | 0.04864405 |
| SCP_left | 0.16235794 | 0 | 3.27838142 |
| SCP_right | 0.0083612 | 0 | 1.94816054 |
| SLF_III_left | 0 | 0 | 0 |
| SLF_III_right | 0 | 0 | 0.5898198 |
| SLF_II_left | 0 | 0 | 0 |
| SLF_II_right | 0 | 0 | 0.00259747 |
| SLF_I_left | 0 | 0 | 0 |
| SLF_I_right | 0 | 0 | 0 |
| ST_FO_left | 0.7240465 | 0 | 0 |
| ST_FO_right | 0 | 0 | 0 |
| ST_OCC_left | 2.14097826 | 0.18979123 | 0 |
| ST_OCC_right | 1.96662694 | 0 | 0.11174017 |
| ST_PAR_left | 1.08489439 | 0.02529547 | 0.00702652 |
| ST_PAR_right | 1.25210047 | 0 | 0.00594822 |
| ST_POSTC_left | 0.4245192 | 0 | 0.21869171 |
| ST_POSTC_right | 0.24101746 | 0 | 0.11494679 |
| ST_PREC_left | 0.47799192 | 0 | 0.5853484 |
| ST_PREC_right | 0.31673156 | 0.20631138 | 0.48236183 |
| ST_PREF_left | 0.69912415 | 0 | 0.41364846 |
| ST_PREF_right | 0.32690489 | 0 | 0.10074427 |
| ST_PREM_left | 0.11767475 | 0 | 0.15297717 |
| ST_PREM_right | 0.13247426 | 0 | 0.0421509 |
| STR_left | 1.03162295 | 0 | 4.53104983 |
| STR_right | 0.82223962 | 0 | 2.11433046 |
| T_OCC_left | 2.96535869 | 0.05455325 | 0.05065659 |
| T_OCC_right | 2.66672703 | 0.03169285 | 0.02263775 |
| T_PAR_left | 1.39180672 | 0 | 0.41641657 |
| T_PAR_right | 1.51807043 | 0.00963243 | 0.53363643 |
| T_POSTC_left | 0.58660361 | 0 | 1.718885 |
| T_POSTC_right | 0.4646283 | 0 | 1.54376499 |
| T_PREC_left | 0.65605117 | 0 | 2.47914812 |
| T_PREC_right | 0.4547217 | 0.30667278 | 1.41703973 |
| T_PREF_left | 1.68235068 | 0.10754753 | 1.38467448 |
| T_PREF_right | 1.3351107 | 0.09164566 | 0.67067964 |
| T_PREM_left | 0.82100089 | 0 | 2.65125765 |
| T_PREM_right | 0.51017579 | 0.06654467 | 1.73570676 |
| UF_left | 0 | 0 | 0 |
| UF_right | 0 | 0 | 0 |

Supplemental Table: Regions Negatively associated with age. Table of all tracts normed to size of tract (% of tract with significant streamlines).

Supplemental Material 4: Regions Positively associated with age. Table of all tracts normed to size of tract (% of tract with significant streamlines).


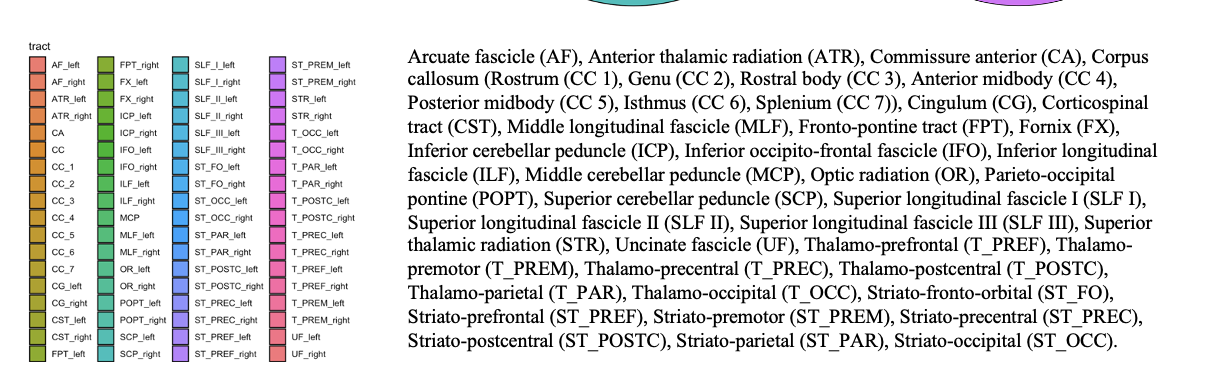


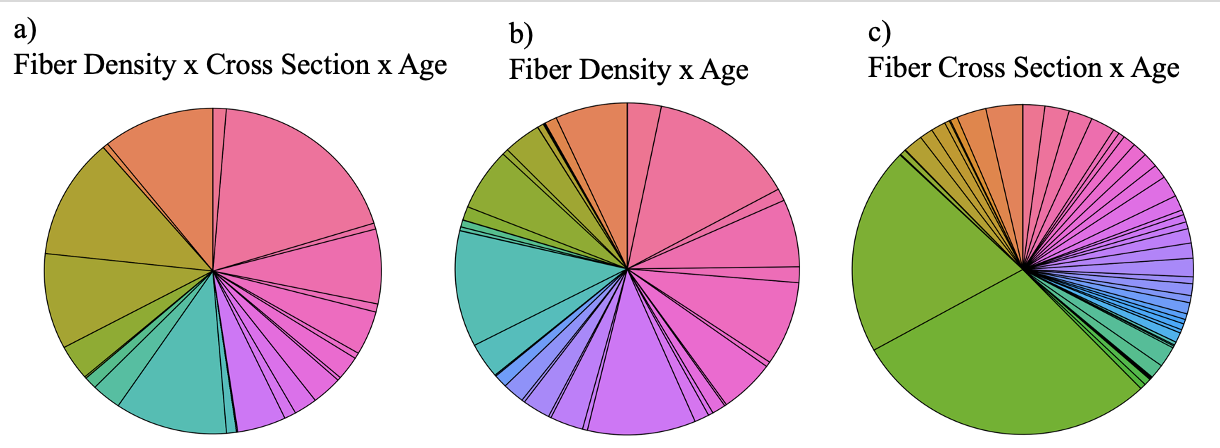


| Tracts | FD x Age (% of tract) | FDC x Age (% of tract) | FC x Age (% of tract) |
| --- | --- | --- | --- |
| AF_left | 0 | 0 | 0 |
| AF_right | 0 | 0 | 0.01685488 |
| ATR_left | 8.07918829 | 0.1865998 | 5.8312438 |
| ATR_right | 1.36219444 | 0.0093301 | 4.69303975 |
| CA | 0 | 0 | 0 |
| CC_1 | 0 | 0 | 0 |
| CC_2 | 0 | 0 | 0.20704948 |
| CC_3 | 0.1191031 | 0 | 0 |
| CC_4 | 0.06089811 | 0 | 0.78902775 |
| CC_5 | 0 | 0 | 2.31926028 |
| CC_6 | 0 | 0 | 2.10840448 |
| CC_7 | 0 | 0 | 3.12941453 |
| CC | 0.05952406 | 0 | 1.142862 |
| CG_left | 0.69625437 | 0.20850398 | 0 |
| CG_right | 0.02116581 | 0.16509334 | 0 |
| CST_left | 4.20677508 | 0 | 0.09830828 |
| CST_right | 0.68705882 | 0 | 0.00941176 |
| FPT_left | 7.2210176 | 0.06051132 | 0.92027634 |
| FPT_right | 1.58832963 | 0.00308414 | 0.14803849 |
| FX_left | 0 | 0 | 34.0631365 |
| FX_right | 0 | 0 | 48.6342944 |
| ICP_left | 0 | 0 | 0 |
| ICP_right | 0 | 0 | 0 |
| IFO_left | 0.01719284 | 0 | 0.92841332 |
| IFO_right | 0 | 0 | 1.09509834 |
| ILF_left | 0 | 0 | 0.04910084 |
| ILF_right | 0 | 0 | 0.1717094 |
| MCP | 0 | 0 | 0 |
| MLF_left | 0 | 0 | 0.16061615 |
| MLF_right | 0 | 0 | 0.14657384 |
| OR_left | 0.80648584 | 0.02122331 | 2.34305361 |
| OR_right | 0 | 0 | 3.39871918 |
| POPT_left | 0.4305427 | 0.04951241 | 0.32075431 |
| POPT_right | 0.02675423 | 0 | 0.2018728 |
| SCP_left | 13.4694642 | 0.18733608 | 0.52454103 |
| SCP_right | 4.10535117 | 0.01672241 | 0 |
| SLF_III_left | 0 | 0 | 0 |
| SLF_III_right | 0 | 0 | 0 |
| SLF_II_left | 0 | 0 | 0 |
| SLF_II_right | 0 | 0 | 0 |
| SLF_I_left | 0 | 0 | 0 |
| SLF_I_right | 0 | 0 | 0 |
| ST_FO_left | 0 | 0 | 1.61125841 |
| ST_FO_right | 0 | 0 | 0.01128286 |
| ST_OCC_left | 0 | 0 | 0.55605501 |
| ST_OCC_right | 0 | 0 | 1.00193683 |
| ST_PAR_left | 0.13912506 | 0 | 0.75043213 |
| ST_PAR_right | 0 | 0 | 0.95022826 |
| ST_POSTC_left | 1.51797774 | 0 | 1.84601531 |
| ST_POSTC_right | 0 | 0 | 1.17171567 |
| ST_PREC_left | 2.4640867 | 0 | 1.94008486 |
| ST_PREC_right | 0.3457895 | 0 | 1.11873075 |
| ST_PREF_left | 3.12663857 | 0.00194201 | 3.01982794 |
| ST_PREF_right | 0.29812081 | 0 | 2.48982277 |
| ST_PREM_left | 3.71852201 | 0 | 2.35349494 |
| ST_PREM_right | 0.55398326 | 0 | 1.14409586 |
| STR_left | 12.1165127 | 0.0809116 | 1.22715933 |
| STR_right | 1.64447925 | 0.01957713 | 0.78961107 |
| T_OCC_left | 0.5533258 | 0.03896661 | 2.49775942 |
| T_OCC_right | 0 | 0 | 3.50885136 |
| T_PAR_left | 1.53061224 | 0.05064526 | 2.29779412 |
| T_PAR_right | 0.2234723 | 0.00577946 | 2.39269477 |
| T_POSTC_left | 6.24346323 | 0.04092583 | 2.34641444 |
| T_POSTC_right | 0.59452438 | 0.00999201 | 2.0333733 |
| T_PREC_left | 9.72867834 | 0.07582501 | 0.99561534 |
| T_PREC_right | 1.82593676 | 0.0140999 | 0.98699284 |
| T_PREF_left | 7.83368542 | 0.12867294 | 3.86402919 |
| T_PREF_right | 1.41842494 | 0.01041428 | 3.75538939 |
| T_PREM_left | 16.493228 | 0.32944622 | 3.86445641 |
| T_PREM_right | 3.78750069 | 0.02218156 | 3.47695891 |
| UF_left | 0 | 0 | 0 |
| UF_right | 0 | 0 | 0 |

Supplemental Table 2: Regions Positively associated with age. Table of all tracts normed to size of tract (% of tract with significant streamlines).

Supplemental material 5: All tracts and age

Supplemental Table 6: Summary statistics of the association between FDC, FD, FC from each tract and age.

| Tract | FD Beta, p-value | FC Beta, p-value | FDC Beta, p-value |
| --- | --- | --- | --- |
| ATR_left | -2.26E-04, 0.53 | -1.50E-03, 0.03* | -1.13E-04, 0.84 |
| ATR_right | -3.49E-04, 0.37 | -2.02E-03, <0.01 | -2.73E-04, 0.66 |
| CA | -1.06E-05, <0.01 | 2.86E-04, 0.66 | -2.41E-04, 0.83 |
| CC_2 | -6.76E-05, <0.01 | 5.05E-05, 0.94 | -1.80E-03, 0.02 |
| CC_4 | 4.94E-04, 0.46 | -1.76E-03, 0.126 | -2.49E-03, 0.01 |
| CC_5 | -1.20E-03, <0.01 | -1.11E-03, 0.406 | -1.39E-03, 0.10 |
| CC_6 | -1.38E-03, <0.01 | -3.12E-04, 0.678 | -1.54E-03, 0.04 |
| CC_7 | -2.02E-03, <0.01 | -5.60E-04, 0.529 | -1.97E-03, 0.02 |
| CC | -1.81E-03, <0.01 | -4.19E-04, 0.628 | -1.82E-03, 0.01 |
| CST_left | -1.33E-03, <0.01 | 5.56E-04, 0.484 | -3.40E-04, 0.74 |
| CST_right | -1.06E-03, <0.01 | -2.84E-04, 0.667 | -1.40E-03, 0.19 |
| FPT_left | -9.32E-04, <0.01 | -1.25E-03, 0.122 | 1.90E-04, 0.81 |
| FPT_right | -1.30E-03, <0.01 | -4.29E-04, 0.471 | 6.81E-05, 0.93 |
| FX_left | 1.52E-05, <0.01 | -1.40E-03, 0.0673 | -2.91E-03, 0.00 |
| FX_right | -7.31E-05, <0.01 | -1.88E-03, 0.013* | -3.21E-03, 0.00 |
| IFO_left | 3.65E-04, 0.55 | -8.21E-04, 0.309 | -1.07E-03, 0.12 |
| IFO_right | -6.65E-05, <0.01 | -1.63E-03, 0.04* | -1.56E-03, 0.03 |
| ILF_left | 9.32E-04, 0.10 | -1.07E-03, 0.08 | -9.46E-04, 0.26 |
| ILF_right | 7.49E-04, 0.22 | -9.15E-04, 0.142 | -1.50E-03, 0.26 |
| MLF_left | -4.02E-03, <0.01 | 4.46E-03, <0.001* | -1.13E-03, 0.11 |
| MLF_right | -4.42E-03, <0.01 | 5.59E-03, <0.001* | -1.54E-03, 0.03* |
| OR_left | 6.83E-05, 0.89 | -1.90E-03, 0.01* | -1.77E-03, 0.02 |
| OR_right | -1.24E-04, 0.76 | -1.78E-03, 0.02* | -2.23E-03, 0.00 |
| POPT_left | -2.48E-04, 0.57 | -1.15E-03, 0.07 | -2.31E-04, 0.75 |
| POPT_right | -4.32E-04, 0.35 | -1.58E-03, 0.01* | -9.90E-04, 0.19 |
| SCP_left | 8.23E-05, <0.01 | -1.52E-03, 0.06 | 8.22E-04, 0.23 |
| SCP_right | 2.11E-05, <0.01 | -2.09E-03, 0.01* | 4.69E-04, 0.46 |
| SLF_III_left | -5.74E-05, <0.01 | -2.07E-03, <0.001* | -1.28E-03, 0.06 |
| SLF_III_right | -4.40E-04, 0.30 | -8.77E-04, 0.21 | -1.83E-03, 0.01 |
| ST_FO_left | -7.45E-04, <0.01 | -7.49E-04, 0.27 | 6.06E-04, 0.43 |
| ST_OCC_left | -4.42E-04, 0.33 | -1.70E-03, 0.03* | -1.64E-03, 0.03 |
| ST_OCC_right | -6.71E-04, 0.16 | -1.89E-03, 0.01* | -2.38E-03, 0.00 |
| ST_PAR_left | 2.39E-06, <0.01 | -1.48E-04, 0.82 | -7.91E-04, 0.22 |
| ST_PAR_right | -4.82E-04, 0.35 | -2.46E-04, 0.69 | -1.42E-03, 0.03 |
| ST_POSTC_left | 1.46E-03, <0.01 | -1.45E-03, 0.02* | 1.18E-04, 0.86 |
| ST_POSTC_right | 1.18E-03, <0.01 | -1.62E-03, <0.001* | -8.41E-04, 0.23 |
| ST_PREC_left | -3.47E-04, 0.34 | -1.76E-03, 0.04* | -5.67E-04, 0.23 |
| ST_PREC_right | -7.23E-04, <0.01 | -2.05E-03, 0.01* | -1.50E-03, 0.05 |
| ST_PREF_left | -4.97E-04, 0.22 | -1.63E-03, 0.03* | -2.03E-04, 0.75 |
| ST_PREF_right | -6.03E-04, <0.01 | -1.60E-03, 0.04* | -3.91E-04, 0.56 |
| ST_PREM_left | -2.75E-04, 0.46 | -1.71E-03, 0.05 | -8.76E-04, 0.16 |
| ST_PREM_right | -3.56E-04, 0.37 | -9.14E-04, 0.26 | -9.72E-04, 0.18 |
| STR_left | 5.00E-04, 0.34 | 2.90E-04, 0.734 | 1.30E-04, 0.90 |
| STR_right | 4.46E-04, 0.46 | -9.08E-04, 0.31 | -9.87E-04, 0.32 |
| T_OCC_left | -4.19E-04, 0.37 | -1.79E-03, 0.01* | -1.73E-03, 0.02 |
| T_OCC_right | -7.82E-04, <0.01 | -2.16E-03, <0.01* | -2.19E-03, 0.00 |
| T_PAR_left | -3.71E-04, 0.37 | -4.37E-04, 0.49 | -5.83E-04, 0.37 |
| T_PAR_right | -7.08E-04, 0.10 | -6.04E-04, 0.33 | -1.35E-03, 0.04* |
| T_POSTC_left | 2.58E-04, 0.54 | -1.75E-06, 0.99 | 6.59E-04, 0.38 |
| T_POSTC_right | -2.42E-04, 0.57 | -6.23E-04, 0.39 | -6.87E-04, 0.38 |
| T_PREC_left | 9.46E-05, 0.83 | -7.45E-04, 0.32 | -3.02E-04, 0.72 |
| T_PREC_right | -2.86E-04, 0.53 | -1.66E-03, 0.02* | -1.36E-03, 0.11 |
| T_PREF_left | 3.77E-04, 0.39 | -6.34E-04, 0.31 | -1.47E-04, 0.81 |
| T_PREF_right | 2.24E-04, 0.63 | -6.85E-04, 0.29 | -3.22E-04, 0.62 |
| T_PREM_left | 1.51E-04, 0.73 | -1.50E-03, 0.04* | -7.81E-05, 0.90 |
| T_PREM_right | -3.06E-05, <0.01 | -1.18E-03, 0.12 | -2.01E-04, 0.78 |
| UF_left | 1.13E-03, <0.01 | -1.39E-03, 0.08 | -1.07-03, 0.25 |
| UF_right | 5.10E-04, 0.40 | -1.70E-03, 0.03* | -3.65E-03, 0.69 |

Supplemental Material 7: Positive fixel associations and cognition

Supplemental Table: Positive FBA x Cognitive Results from GLM.

|  | **Fiber Density** | **Fiber Cross Section** | **Fiber Density * Cross Section** |
| --- | --- | --- | --- |
| **Processing Speed** | β=-0.01, p=0.99 | β=-0.44, p=0.10 | β=-0.06, p=0.95 |
| **Visuospatial** | β=-0.52, p=0.39 | β=-0.29, p=0.18 | β=-0.46, p=0.39 |
| **Episodic Memory** | β=0.13, p=0.92 | β=-0.24, p=0.39 | β=-0.55, p=0.39 |
| **Working Memory** | β=-0.43, p=0.39 | β =-0.39, p=0.08 | β=-0.45, p=0.39 |
| **Attentional Control** | β=-0.49, p=0.39 | β=-0.44, p=0.08 | β=-0.28, p=0.18 |

*all p values in this table are FDR corrected

Supplemental Material 8

Supplemental Table: Correlation coefficient between tract based FDC and FA. All tracts are p>0.01.

| Tract | Correlation coefficient |
| --- | --- |
| ATR_left | 0.4926178 |
| ATR_right | 0.4578639 |
| CA | 0.3989272 |
| CC_2 | 0.4081847 |
| CC_4 | 0.466374 |
| CC_5 | 0.3819834 |
| CC_6 | 0.5016793 |
| CC_7 | 0.519537 |
| CC | 0.6272947 |
| CST_left | 0.5846499 |
| CST_right | 0.5453229 |
| FPT_left | 0.5727931 |
| FPT_right | 0.5897104 |
| FX_left | 0.415155 |
| FX_right | 0.417276 |
| IFO_left | 0.6549319 |
| IFO_right | 0.6265941 |
| ILF_left | 0.563139 |
| ILF_right | 0.542651 |
| MLF_left | 0.4888444 |
| MLF_right | 0.4883942 |
| OR_left | 0.4574518 |
| OR_right | 0.4712735 |
| POPT_left | 0.5304062 |
| POPT_right | 0.5328455 |
| SCP_left | 0.4271537 |
| SCP_right | 0.3992378 |
| SLF_III_left | 0.4833653 |
| SLF_III_right | 0.4757967 |
| ST_FO_left | 0.4705803 |
| ST_OCC_left | 0.5043128 |
| ST_OCC_right | 0.5311761 |
| ST_PAR_left | 0.5665995 |
| ST_PAR_right | 0.5491642 |
| ST_POSTC_left | 0.5369788 |
| ST_POSTC_right | 0.5485647 |
| ST_PREC_left | 0.4843068 |
| ST_PREC_right | 0.4543097 |
| ST_PREF_left | 0.3923862 |
| ST_PREF_right | 0.3563216 |
| ST_PREM_left | 0.3955441 |
| ST_PREM_right | 0.3899278 |
| STR_left | 0.4549097 |
| STR_right | 0.5179924 |
| T_OCC_left | 0.5286268 |
| T_OCC_right | 0.5472335 |
| T_PAR_left | 0.5351115 |
| T_PAR_right | 0.5110325 |
| T_POSTC_left | 0.5701916 |
| T_POSTC_right | 0.536303 |
| T_PREC_left | 0.584203 |
| T_PREC_right | 0.5616718 |
| T_PREF_left | 0.4867143 |
| T_PREF_right | 0.4795199 |
| T_PREM_left | 0.4646269 |
| T_PREM_right | 0.4667822 |
| UF_left | 0.5691634 |
| UF_right | 0.5439421 |
